# Transformation of (allo)securinine to (allo)norsecurinine via a molecular editing strategy

**DOI:** 10.3389/fchem.2024.1355636

**Published:** 2024-01-22

**Authors:** Seoyoung Kim, Hee-Seung Lee, Sunkyu Han

**Affiliations:** Department of Chemistry, Korea Advanced Institute of Science & Technology (KAIST), Daejeon, Republic of Korea

**Keywords:** securinega alkaloids, ring contraction, molecular editing, natural products, atom deletion strategy, heterocycle

## Abstract

Securinega alkaloids have intrigued chemists since the isolation of securinine in 1956. This family of natural products comprises a securinane subfamily with a piperidine substructure and norsecurinane alkaloids featuring a pyrrolidine core. From a biosynthetic perspective, the piperidine moiety in securinane alkaloids derives from lysine, whereas the pyrrolidine moiety in norsecurinane natural products originates from ornithine, marking an early biogenetic divergence. Herein, we introduce a single-atom deletion strategy that enables the late-stage conversion of securinane to norsecurinane alkaloids. Notably, for the first time, this method enabled the transformation of piperidine-based (allo)securinine into pyrrolidine-based (allo)norsecurinine. Straightforward access to norsecurinine from securinine, which can be readily extracted from the plant *Flueggea suffruticosa*, abundant across the Korean peninsula, holds promise for synthetic studies of norsecurinine-based oligomeric securinega alkaloids.

## Introduction

**SCHEME 1:**
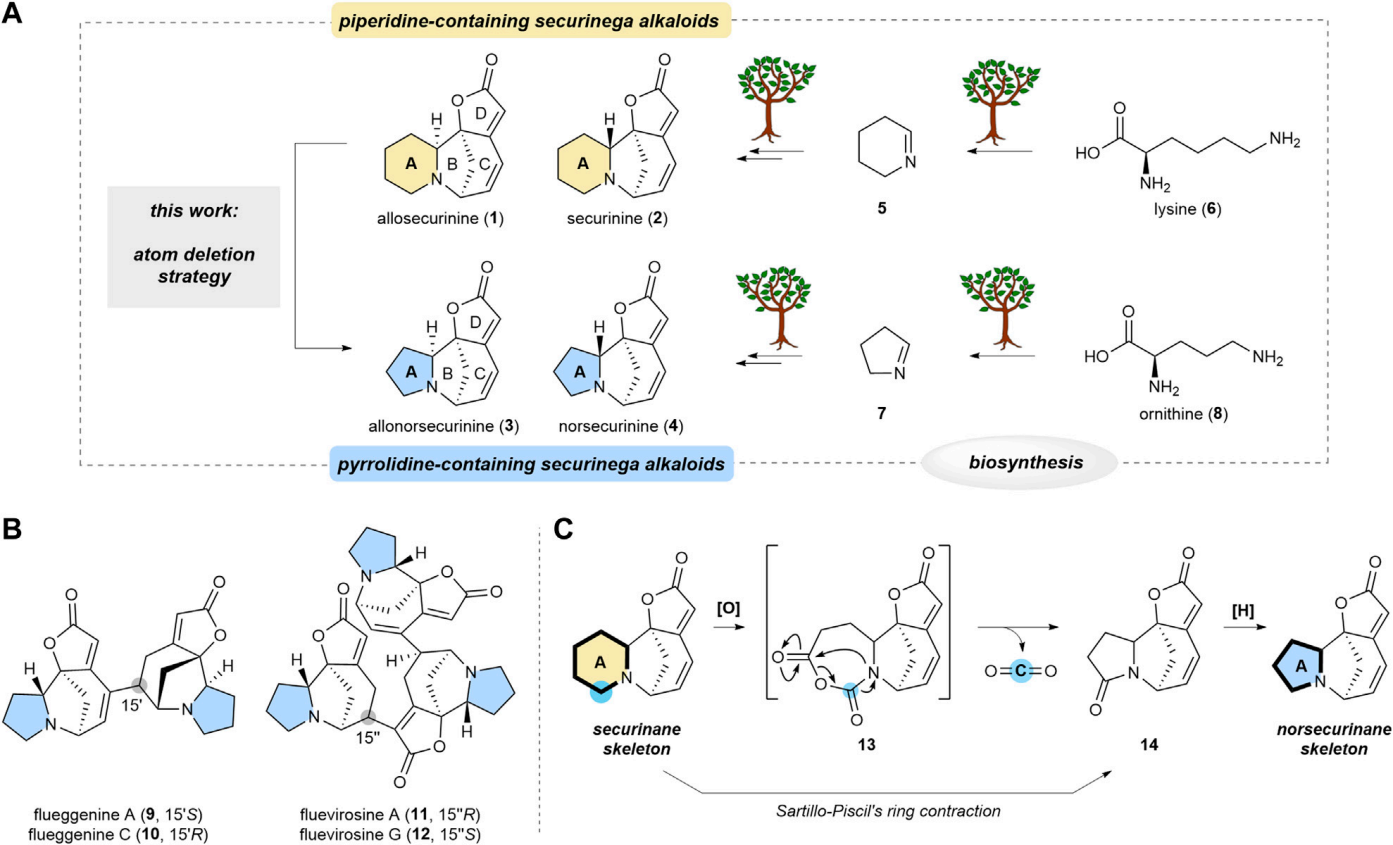
Transformation of securinane framework to norsecurinane framework via molecular editing strategy. **(A)** Parallel biosynthesis of (allo)securinine and (allo)norsecurinine and the direct transformation of the former natural products to the latter natural product (this work). **(B)** Representative high-order securinega alkaloids with the pyrrolidine core. **(C)** Overview of our atom deletion strategy that is based on Sartillo-Piscil’s ring contraction method.

Securinega alkaloids, composed of over 100 members, have fascinated the synthetic community owing to their structural diversity (Wehlauch and Gademann, 2017; Kang et al., 2023) and potent bioactivities (Hou et al., 2023) since the isolation of securinine (**2**) in 1956 (Muraveva and Bankovskii, 1956). Securinane alkaloids, exemplified by allosecurinine (**1**) and securinine (**2**), are characterized by an azabicyclo[3.2.1]octane core (B/C rings), fused with a piperidine (A) and butenolide (D) rings (Scheme 1A). Norsecurinane alkaloids such as allonorsecurinine (**3**) (Alibés et al., 2004; Antien et al., 2019; Hetzler et al., 2022) and norsecurinine (**4**) share a structural resemblance with securinanes. However, they feature a pyrrolidine moiety in place of the piperidine-based A ring. It is established that the piperidine (A) ring of the securinane alkaloids is derived from 1-piperideine (**5**), the biosynthetic downstream derivative of lysine (**6**) (Marek Golebiewski et al., 1976). On the other hand, the pyrrolidine (A) ring of the norsecurinane alkaloids is envisioned to have originated from 1-pyrroline (**7**), the biosynthetic derivative of ornithine (**8**). Hence, the size of the A ring is determined at the early stage of the biogenesis.

Notably, various dimeric and oligomeric high-order securinega alkaloids such as flueggenines A (**9**), C (**10**), and fluevirosines A (**11**), G (**12**) have been isolated (Scheme 1B) (Jeon et al., 2017; Kang et al., 2023). In 2017, we reported the total synthesis of flueggenine C (**10**) via an accelerated Rauhut–Currier (RC) reaction (Jeon and Han, 2017). Subsequently, we also succeeded in the total synthesis of flueggenines D and I via a Stille cross-coupling and stereoselective conjugate reduction dimerization strategy (Jeon et al., 2020). Notably, the primary constituent among the monomeric units forming high-order securinega alkaloids is norsecurinine (**4**). In pursuit of a synthetic program centered on direct RC-based conjugations among norsecurinines (**4**) (Han, 2023; Park et al., 2023), we sought a solution for obtaining a substantial quantity of norsecurinine (**4**). In consideration of this goal, we conceived a semisynthetic method to derive (allo)norsecurinine (**3** and **4**) from (allo)securinine (**1** and **2**), natural substances abundantly found in *Flueggea suffruticosa*, a widely distributed plant in the Korean peninsula.

Single-atom skeletal editing has emerged as a powerful approach to efficiently diversify the molecular framework (Jurczyk et al., 2022). This molecular editing strategy has also been applied in natural product synthesis (Hui et al., 2022). We envisioned that the securinane skeleton could be transformed into the norsecurinane skeleton via a single-atom deletion logic (Ha, 2023). Herein, we describe a synthetic protocol that enables a single carbon deletion of the piperidine (A) (Srivastava and Ha, 2023) ring of securinanes to yield pyrrolidine-based norsecurinanes via the intermediacy of *N*-carboxyanhydride intermediate **13** and lactam **14** (Scheme 1C) based on Sartillo-Piscil’s ring contraction method (Romero-Ibañez et al., 2019).

## Results and discussion

**SCHEME 2:**
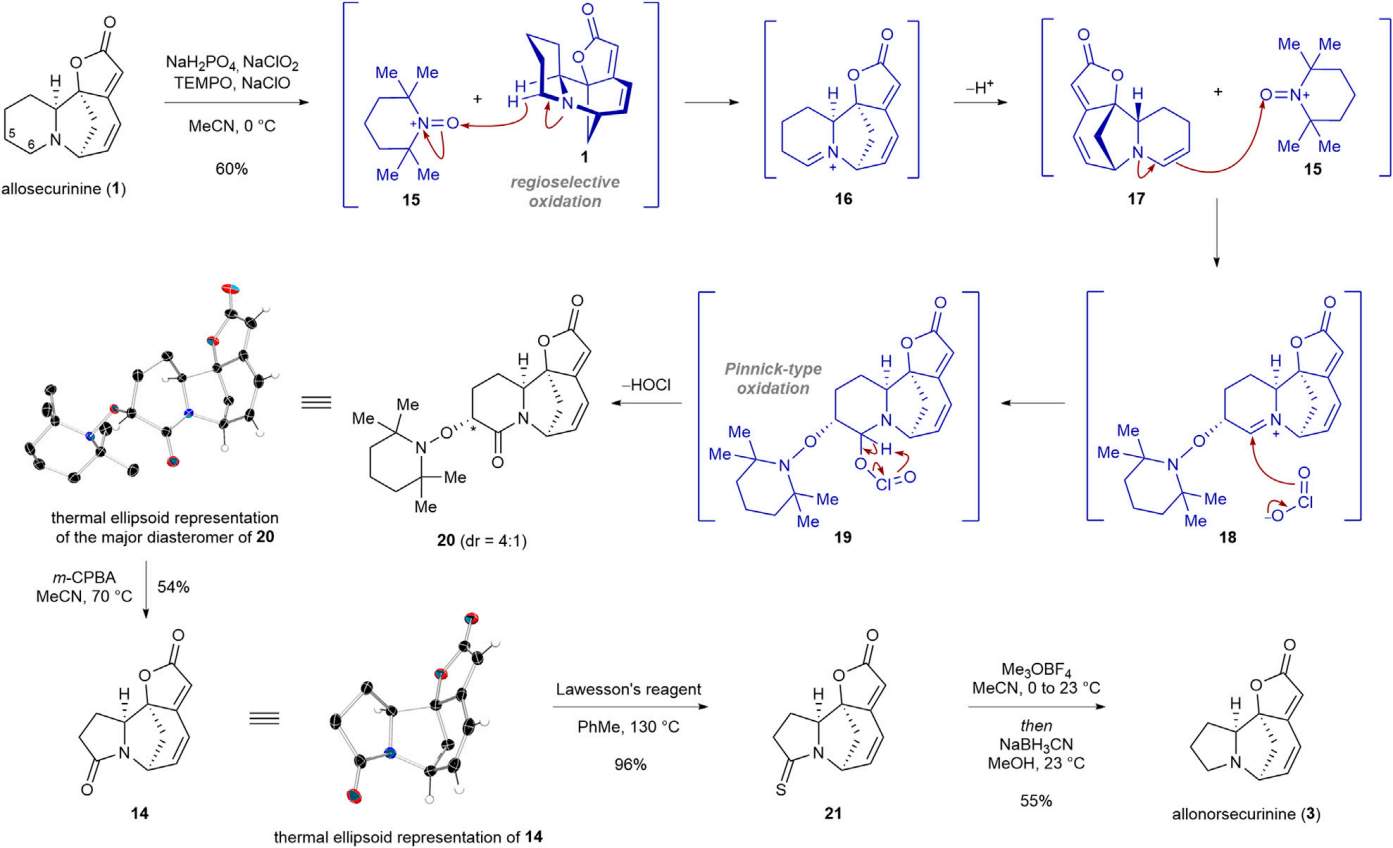
Transformation of allosecurinine (**1**) to allonorsecurinine (**3**) via an atom deletion strategy.

Our synthesis commenced with regioselective double oxidation of C5 and C6 carbons of allosecurinine (**1**) to produce lactam **20** in 60% yield as a 4:1 diastereomeric mixture (Scheme 2). In 2016, Sartillo-Piscil and coworkers reported a double sp^3^ C−H oxidation of cyclic amines to α-alkoxyamine lactams (Osorio-Nieto et al., 2016; Romero-Ibañez et al., 2023). In the instance of allosecurinine (**1**), the oxoammonium cation of TEMPO (**15**) selectively abstracted the C6 hydridic α-hydrogen from the amine moiety due to its greater steric accessibility consistent with Sartillo-Piscil’s reports (Osorio-Nieto et al., 2016; Romero-Ibañez et al., 2023). This led to the formation of the iminium ion intermediate **16**, which subsequently underwent tautomerization, resulting in the formation of enamine **17**. Subsequently, enamine **17** reacted with the oxoammonium cation of TEMPO (**15**) to produce iminium ion **18**, which is consequently trapped by the chlorine dioxide anion to afford intermediate **19** (Osorio-Nieto et al., 2016). The extrusion of hypochlorous acid from intermediate **19** resulted in lactam **20**. The structure of the major diastereomer of **20** was unambiguously confirmed by a single crystal X-ray diffraction (SCXD) analysis. To our delight, when the TEMPO adduct **20** was allowed to react with 3 equiv of *m*-CPBA, the ring contraction product **14** was obtained in 54% yield (Romero-Ibañez et al., 2019). The structure of lactam **14** was confirmed by a single crystal X-ray diffraction (SCXD) analysis. Chemoselective reduction of the lactam moiety of **14** in the presence of the lactone group was achieved by a synthetic sequence involving the conversion of the lactam moiety to the thiolactam group (96% yield), the methylation of the resulting thiolactam moiety, and the sodium cyanoborohydride-mediated reduction of the consequent thioimidate ester salt (55% yield for the conversion of **21** to **3**).

**SCHEME 3:**
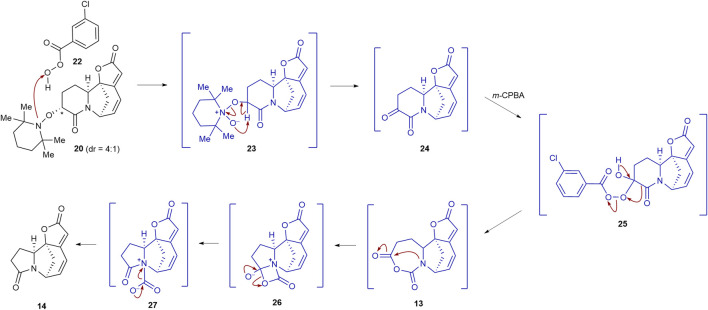
Plausible reaction mechanism for the formation of **14** from **20** in the presence of *m*-CPBA.

The plausible reaction mechanism for converting TEMPO adduct **20** to lactam **14** is depicted in Scheme 3 (Romero-Ibañez et al., 2019). The *m*-CPBA-mediated oxidation of TEMPO adduct **20** would result in the formation of *N*-oxide intermediate **23**. Extrusion of TEMPOH from **23** would afford α-ketolactam intermediate **24**. Ketone **24** would then undergo a regioselective Baeyer–Villiger oxidation via intermediate **25** to produce *N*-carboxyanhydride intermediate **13**. Transannular 1,2-addition of the nitrogen nucleophile in **13** would yield pentacyclic intermediate **26**, which subsequently undergoes a carbon dioxide extrusion via intermediate **27** to forge lactam **14**.

**SCHEME 4:**
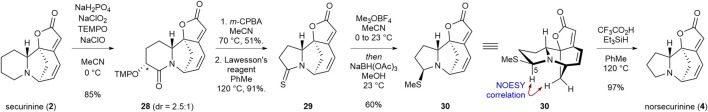
Transformation of securinine (**2**) to norsecurinine (**4**).

After establishing a synthetic protocol for the “methylene deletion” of allosecurinine (**1**), we set out to transform securinine (**2**) to norsecurinine (**4**) in an analogous manner. In the event, treatment of securinine (**2**) with monosodium phosphate, sodium chlorite, TEMPO, sodium hypochlorite in acetonitrile resulted in TEMPO adduct **28** in 85% yield as a 2.5:1 mixture of diastereomers (Scheme 4). Subsequent *m*-CPBA-mediated ring contraction (51% yield) and the thionylation of the resulting lactam with Lawesson’s reagent (91%) afforded thiolactam **29**. Interestingly, when thiolactam **29** was allowed to react with Meerwein’s salt and subsequently with sodium triacetoxyborohydride, thioaminal product **30** was obtained in 60% yield. This was in stark contrast to the aforementioned reduction of thioamide **21** to the amine product under analogous reduction conditions. 2D-NMR analysis of compound **30** revealed that the proton at C5 is axially positioned. Hence, we reasoned that the stereoelectronic misalignment of the N1-lone pair p-orbital and the C5–S σ* orbital hindered the formation of the iminium ion intermediate. To our pleasure, we discovered that exposure of thioaminal product **30** to more forcing conditions (TFA and triethylsilane in refluxing toluene) afforded norsecurinine (**4**) in 97% yield.

## Conclusion

In summary, a molecular editing protocol for the transformation of (allo)securinine to (allo)norsecurinine was established. The key to our success was the employment of the oxidative ring contraction method pioneered by the Sartillo-Piscil group (Romero-Ibañez et al., 2019). Chemoselective reduction of the lactam moiety over the lactone moiety was possible via selective conversion of the amide group to the thioamide group and its subsequent chemoselective reduction. Our findings enabled a straightforward transformation of securinine into norsecurinine, leveraging the abundant supply of securinine derived from the *F. suffruticosa* plant commonly found across the Korean peninsula. Norsecurinine, a pyrrolidine-based compound, serves as the monomeric unit within the high-order securinega alkaloids. Hence, our discovery stands poised to enable the synthesis of more complex securinega alkaloids. Those studies are currently ongoing and will be the subject of forthcoming reports.

## Materials and methods

### General information

All reactions were performed in oven-dried or flame-dried round-bottomed flasks and vials. Unless otherwise noted, the flasks were fitted with rubber septa and reactions were conducted under a positive pressure of argon, and vials were tightly sealed with plastic septa, Teflon tape, and parafilm. Stainless steel syringes were used to transfer air- and moisture-sensitive liquids. Flash column chromatography was performed as described by Still et al. using silica gel (60-Å pore size, 40–63 μm, 4%–6% H_2_O content, Merck) (Still et al., 1978). Analytical thin–layer chromatography (TLC) was performed using glass plates pre-coated with 0.25 mm silica gel impregnated with a fluorescent indicator (254 nm). Thin layer chromatography plates were visualized by exposure to ultraviolet light, and/or a basic aqueous potassium permanganate (KMnO_4_).

Unless otherwise stated, all commercial reagents and solvents were used without additional purification.

^1^H and ^13^C nuclear magnetic resonance spectra were recorded with Bruker Avance Ⅲ HD (400 MHz), Bruker Avance NEO (500 MHz), Agilent DD-2 (600 MHz) and calibrated by using the residual undeuterated chloroform (δ_H_ = 7.24 ppm) and CDCl_3_ (δ_C_ = 77.23 ppm) as internal references. Data are reported in the following manners: chemical shift in ppm [multiplicity (s = singlet, d = doublet, t = triplet, q = quartet, p = quintet, m = multiplet, app = apparent, br = broad), coupling constant(s) in Hertz, integration]. The NMR solvent CDCl_3_ was taken from a stock containing anhydrous K_2_CO_3_ to remove residual DCl. High resolution mass spectra were obtained from KAIST Analysis Center for Research (Daejeon) by using ESI method. Specific rotation ${\left[\alpha \right]}_{D}^{T}$ was obtained by JASCO P-2000 polarimeter.

## Data Availability

The original contributions presented in the study are included in the article/Supplementary Material, further inquiries can be directed to the corresponding author. Single crystal X-ray diffraction analysis data of compounds **14** (deposition number: 2314385) and **20** (deposition number: 2314384) were deposited in the Cambridge Crystallographic Data Center (CCDC).
