# Supplementary material for: Transformation of (allo)securinine to (allo)norsecurinine via a molecular editing strategy
Source: Front Chem. 2024 Jan 22;12:1355636. doi: 10.3389/fchem.2024.1355636 (PMC10839145; doi:10.3389/fchem.2024.1355636)

Transformation of (allo)securinine to (allo)norsecurinine via a molecular editing strategy

Seoyoung Kim, Hee-Seung Lee and Sunkyu Han*
Department of Chemistry, Korea Advanced Institute of Science and Technology (KAIST), Daejeon 34141, Republic of Korea

Supplementary Information

1. General Information S02
2. Isolation of allosecurinine (**1**) and securinine (**2**) from the Roots of *Flueggea suffruticosa* S03
3. Experimental Procedures and Physical Data for Synthesized Compounds S04
4. Comparison of Spectroscopic Data of Synthetic Natural Products with References S15
  4.1. NMR data of previous synthetic and present synthetic allonorsecurinine (**3**) S15
  4.2. NMR data of previous synthetic and present synthetic norsecurinine (**4**) S17
5. Single Crystal X-Ray Diffraction Analysis of Compounds **20** and **14** S19
  5.1. X-ray Crystal Structure of α-alkoxyamine lactam **20** (major diastereomer) S19
  5.2. X-ray Crystal Structure of 5-oxoallonorsecurinine (**14**) S29
6. Supplemental References S37
7. Copies of NMR spectra of newly Synthesized Compounds S38

1. General Information

All reactions were performed in oven-dried or flame-dried round-bottomed flasks and vials. Unless otherwise noted, the flasks were fitted with rubber septa and reactions were conducted under a positive pressure of argon, and vials were tightly sealed with plastic septa, Teflon tape, and parafilm. Stainless steel syringes were used to transfer air- and moisture-sensitive liquids. Flash column chromatography was performed as described by Still et al. using silica gel (60-Å pore size, 40–63 μm, 4-6% H_2O content, Merck).[1] Analytical thin–layer chromatography (TLC) was performed using glass plates pre-coated with 0.25 mm silica gel impregnated with a fluorescent indicator (254 nm). Thin layer chromatography plates were visualized by exposure to ultraviolet light, and/or a basic aqueous potassium permanganate ($KMnO_4$).

Unless otherwise stated, all commercial reagents and solvents were used without additional purification with the following exceptions as indicated below.

^{1}H and ^{13}C nuclear magnetic resonance spectra were recorded with Bruker Avance III HD (400 MHz), Bruker Avance NEO (500 MHz), Agilent DD-2 (600 MHz) and calibrated by using the residual undeuterated chloroform (δ_H= 7.24 ppm) and $CDCl_3$ (δ_C= 77.23 ppm) as internal references. Data are reported in the following manners: chemical shift in ppm [multiplicity (s = singlet, d = doublet, t = triplet, q = quartet, p = quintet, m = multiplet, app = apparent, br = broad), coupling constant(s) in Hertz, integration]. The NMR solvent $CDCl_3$ was taken from a stock containing anhydrous K_2CO_3 to remove residual DCl. High resolution mass spectra were obtained from KAIST Analysis Center for Research (Daejeon) by using ESI method. Specific rotation $[\alpha]_D^T$ was obtained by JASCO P-2000 polarimeter.

2. Isolation of allosecurinine (1) and securinine (2) from the Roots of *Flueggea suffruticosa*

1.0 kg of dried roots were cut into pieces, placed in 5 L glass bottle, and 1,2-dichloroethane (4 L), 10% aqueous ammonium hydroxide (400 mL) was added to the bottle. The bottle was sealed and stirred at 23 °C. After 36 h, the resulting yellow mixture was filtered, dried over anhydrous sodium sulfate. The resulting filtrate was concentrated under reduced pressure. The extraction process was repeated three times. The resulting crude residue was purified by iterative flash column chromatography (silica gel: diam: 8 cm, ht = 12 cm; eluent: diethyl ether (1% NH_4OH)) to yield allosecurinine (**1**, overall 5.2 g, calculated 0.52% from the root material) as a yellow powder. Also fraction of securinine crude collected from previous flash column chromatography, purified by iterative flash column chromatography (silica gel: diam: 4 cm, ht = 19 cm; eluent: diethyl ether : hexane = 1 : 1) to yield securinine (**2**, overall 1.2 g, calculated 0.12% from the root material) as a yellow powder.

Note: The isolation protocol was originally reported in J. Am. Chem. Soc. ***2022****, 144, 8932–8937.* [2]

3. Experimental Procedures and Physical Data for Synthesized Compounds

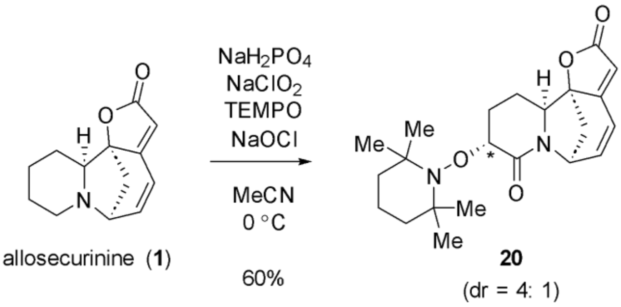

α-Alkoxyamine lactam 20:

Sodium hypochlorite solution (aq, 3%, 1.84 mL, 0.368 mmol, 1.0 equiv.) was added dropwise to a suspension of sodium phosphate monobasic anhydride (441.8 mg, 3.682 mmol, 10.0 equiv.), sodium chlorite (100.0 mg, 1.104 mmol, 3.0 equiv.) and TEMPO (115.0 mg, 0.736 mmol, 2.0 equiv.) in acetonitrile (6.14 mL, 0.06 M) at 0 °C under argon atmosphere and stirred at 0 °C for 5 min. Then, a solution of allosecurinine (**1**, 80.0 mg, 0.368 mmol, 1.0 equiv.) in acetonitrile (1.2 mL, 0.3 M) was added dropwise over 10 min to an aforementioned suspension and the resulting mixture was further stirred at 0 °C for 1 h. The resulting reaction mixture diluted with ethyl acetate (20 mL) and poured over saturated aqueous sodium thiosulfate solution (20 mL) in separatory funnel. Sodium hydroxide (aq, 3 M) was added dropwise to the mixture until pale yellow precipitates were formed. Subsequently, brine was added to redissolve the precipitates, and the aqueous layer was extracted with ethyl acetate (3 × 40 mL). Combined organic layer was dried over anhydrous sodium sulfate and concentrated under reduced pressure. The resulting crude residue was purified by flash column chromatography (silica gel: diam: 3 cm, ht: 15 cm, eluent: acetone : hexanes = 1 : 4) to afford **20** (85.7 mg, 60%, dr = 4 : 1) as a white powder. Analytic samples of major and minor diastereomers of **20** were isolated via iterative flash column chromatography (silica gel: diam: 3 cm, ht: 20 cm, eluent: acetone : hexanes = 1 : 4).

Note: Crystals of **20** (major product) were obtained by a slow evaporation of the dichloromethane solution as a colorless needle.

α-Alkoxyamine lactam 20 (major diastereomer)

^{1}H NMR (400 MHz, $CDCl_3$): δ 7.05 (dd, *J* = 8.9, 5.6 Hz, 1H), 6.50 (d, *J* = 8.9 Hz, 1H), 5.86 (s, 1H), 4.77 (t, *J* = 5.3 Hz, 1H), 4.39 (dd, *J* = 9.7, 5.9 Hz, 1H), 4.19 (t, *J* = 4.3 Hz, 1H), 2.62 (dd, *J* = 10.3, 5.0 Hz, 1H), 2.16 – 2.04 (m, 2H), 1.95 – 1.81 (m, 1H), 1.81 – 1.70 (m, 1H), 1.54 – 1.37 (br, 5H), 1.21 – 1.01 (br, 14H).

^{13}C NMR (101 MHz, $CDCl_3$): δ 171.6, 169.6, 165.0, 145.9, 122.4, 111.6, 90.0, 82.2, 60.8, 59.7, 59.2, 52.0, 42.4, 40.2, 40.1, 33.6, 33.2, 26.0, 21.1, 20.5, 20.2, 17.3.

HRMS (ESI): Calculated for $C_{22}H_{30}N_2O_4$ $[M+H]^+$: 387.2278, found: 387.2281.

TLC (acetone : hexanes = 1 : 1) R*f*: 0.70 (UV, $KMnO_4$).

$[\alpha]_D^{25}$**:** –387.2 (c 0.1, EtOH)

<u>α-Alkoxyamine lactam 20 (minor diastereomer)</u>

^{1}H NMR (400 MHz, $CDCl_3$): δ 7.08 (dd, *J* = 9.0, 5.6 Hz, 1H), 6.50 (dd, *J* = 9.0, 1.0 Hz, 1H), 5.83 (s, 1H), 4.76 (t, *J* = 5.2 Hz, 1H), 4.37 (t, *J* = 8.2 Hz, 1H), 4.04 (dd, *J* = 11.0, 4.5 Hz, 1H), 2.63 (dd, *J* = 10.4, 5.0 Hz, 1H), 2.32 (dq, *J* = 13.7, 8.3 Hz, 1H), 2.08 (d, *J* = 10.3 Hz, 1H), 1.86 (dtd, *J* = 13.7, 8.5, 3.9 Hz, 1H), 1.74 (ddt, *J* = 12.4, 8.2, 4.2 Hz, 1H), 1.43 – 1.39 (br, *J* = 4.0 Hz, 4H), 1.30 – 1.04 (br, 15H).

^{13}C NMR (101 MHz, $CDCl_3$): δ 171.6, 170.1, 165.0, 146.2, 122.0, 111.3, 89.9, 80.3, 61.5, 59.9, 59.5, 52.6, 42.3, 40.8, 35.0, 33.1, 27.3, 22.2, 20.7, 20.4, 17.3.

HRMS (ESI): Calculated for $C_{22}H_{30}N_2O_4$ $[M+H]^+$: 387.2278, found: 387.2281.

TLC (acetone : hexanes = 1 : 1) R*f*: 0.78 (UV, $KMnO_4$).

$[\alpha]_D^{25}$: –295.3 (c 0.1, EtOH)

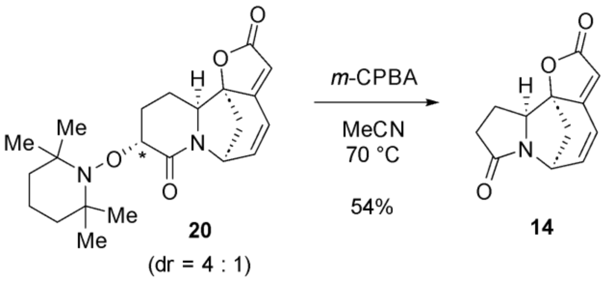

5-Oxoallonorsecurinine (14):

meta-Chloroperbenzoic acid (77% purity, 149.1 mg, 0.665 mmol, 3.0 equiv.) in acetonitrile (2 mL) was added dropwise to a solution of **20** (85.7 mg, 0.222 mmol, 1.0 equiv.) in acetonitrile (15.8 mL, 0.014 M) at 70 °C under argon atmosphere and stirred at 70 °C. After 24 h, the resulting reaction mixture was poured over saturated aqueous sodium thiosulfate solution (30 mL) and stirred 20 min. After 20 min, saturated aqueous sodium bicarbonate solution (30 mL) was poured and extracted with ethyl acetate (3 × 50 mL). Combined organic layer was dried over anhydrous sodium sulfate and concentrated under reduced pressure. The resulting crude residue was purified by flash column chromatography (silica gel: diam: 3 cm, ht: 16 cm; eluent: acetone : hexanes = 1 : 1) to afford 5-oxoallonorsecurinine (**14**, 26 mg, 54%) as a pale yellow powder.

Note: Crystals of **14** were obtained by a slow evaporation of the dichloromethane solution as a colorless needle.

^{1}H NMR (400 MHz, $CDCl_3$): δ 7.20 (dd, *J* = 9.0, 5.3 Hz, 1H), 6.57 (dd, *J* = 8.9, 1.1 Hz, 1H), 5.90 (s, 1H), 4.55 (dd, *J* = 9.5, 6.4 Hz, 1H), 4.47 (t, *J* = 5.1 Hz, 1H), 2.89 (dd, *J* = 10.5, 4.9 Hz, 1H), 2.75 (ddd, *J* = 16.5, 13.2, 8.4 Hz, 1H), 2.27 (dd, *J* = 16.5, 8.9 Hz, 1H), 2.18 (d, *J* = 10.4 Hz, 1H), 2.10 (ddd, *J* = 12.6, 8.3, 6.4 Hz, 1H), 1.51 (tt, *J* = 13.0, 9.2 Hz, 1H).

^{13}C NMR (101 MHz, $CDCl_3$): δ 177.5, 171.5, 164.4, 148.0, 122.3, 112.2, 88.3, 64.6, 51.6, 46.1, 36.0, 24.5.

HRMS (ESI): Calculated for $C_{12}H_{11}NO_3$ $[M+H]^+$: 218.0812, found: 218.0815.

TLC (acetone : hexanes = 4 : 6) R*f*: 0.44 (UV, $KMnO_4$).

$[\alpha]_D^{25}$: –941.9 (c 0.1, EtOH)

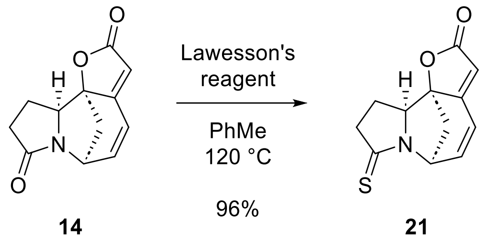

Thiolactam 21:

Lawesson's reagent (37.3 mg, 9.23 ×10^{-2} mmol, 1.2 equiv.) was added to a solution of **14** (16.7 mg, 7.69 ×10^{-2} mmol, 1.0 equiv.) in toluene (0.77 mL, 0.1 M) at 23 °C under argon atmosphere. The resulting mixture was heated to 120 °C. After 30 min, the reaction mixture was diluted with ether (10 mL) and filtered through a pad of celite. Volatiles were evaporated under reduced pressure, and the resulting crude mixture was purified by flash column chromatography (silica gel: diam: 3 cm, ht: 14 cm; eluent: acetone : hexanes = 1 : 4) to afford thiolactam **21** (17.3 mg, 96%) as a white solid.

^{1}H NMR (400 MHz, $CDCl_3$): δ 7.36 (dd, *J* = 9.0, 5.4 Hz, 1H), 6.57 (dd, *J* = 9.0, 1.1 Hz, 1H), 5.93 (s, 1H), 4.86 (t, *J* = 5.3 Hz, 1H), 4.77 (dd, *J* = 10.4, 5.8 Hz, 1H), 3.19 (ddd, *J* = 17.0, 13.2, 7.2 Hz, 1H), 3.01 – 2.90 (m, 2H), 2.28 (d, *J* = 10.7 Hz, 1H), 2.17 (ddd, *J* = 12.6, 7.1, 5.8 Hz, 1H), 1.61 – 1.48 (m, 1H).

^{13}C NMR (101 MHz, $CDCl_3$): δ 203.1, 171.1, 163.6, 146.0, 122.4, 113.0, 87.2, 71.8, 54.3, 49.0, 46.2, 27.2.

HRMS (ESI): Calculated for $C_{12}H_{11}NO_2S$ $[M+H]^+$: 234.0583, found: 234.0584.

TLC (acetone : hexanes = 1 : 4) R*f*: 0.35 (UV, $KMnO_4$).

$[\alpha]_D^{25}$: –1294.3 (c 0.1, EtOH)

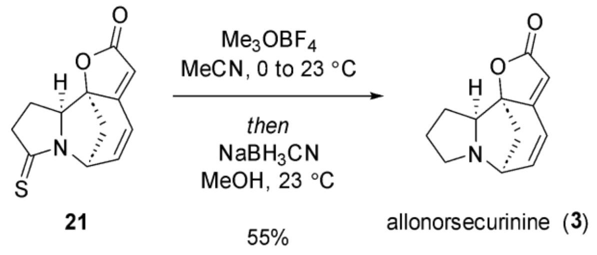

allonorsecurinine (3):

Trimethyloxonium tetrafluoroborate (20.5 mg, 0.139 mmol, 3.0 equiv.) was added to a solution of **21** (10.8 mg, 4.63 $\times 10^{-2}$ mmol, 1.0 equiv.) in acetonitrile (0.5 mL, 0.1 M) at 0 °C under an atmosphere of argon, stirred at 23 °C for 1 h. Volatiles were evaporated in vacuo and the residue was dissolved in methanol (0.5 mL, 0.1 M). Sodium cyanoborohydride (5.8 mg, 9.26 $\times 10^{-2}$ mmol, 2.0 equiv.) was added to the aforementioned solution. After stirring at 23 °C for 2 h, the reaction mixture was poured to a saturated aqueous sodium bicarbonate solution (10 mL) and extracted with dichloromethane (3 × 20 mL). The combined organic layers were dried over anhydrous sodium sulfate. The filtrate was concentrated over reduced pressure, and the resulting crude residue was purified by flash column chromatography (silica gel: diam. 3 cm, ht. 12 cm; eluent: dichloromethane : methanol = 19 : 1) to afford allonorsecurinine (**3**, 5.2 mg, 55%) as a yellow amorphous solid.

^{1}H NMR (500 MHz, $CDCl_3$): δ 6.84 (dd, *J* = 9.1, 5.4 Hz, 1H), 6.68 (dd, *J* = 9.1, 1.1 Hz, 1H), 5.76 (s, 1H), 4.13 (t, *J* = 7.4 Hz, 1H), 3.95 (t, *J* = 4.97 Hz 1H), 2.91 – 2.86 (m, 2H), 2.85 – 2.75 (m, 1H), 2.01 (d, *J* = 9.9 Hz, 1H), 1.85 (tdd, *J* = 12.2, 7.1, 5.1 Hz, 1H), 1.82 – 1.69 (m, 1H), 1.63 (dtd, *J* = 12.6, 7.3, 5.1 Hz, 1H), 1.23 (ddt, *J* = 12.7, 8.5, 7.4 Hz, 1H).

^{13}C NMR (126 MHz, $CDCl_3$): δ 172.5, 167.2, 149.3, 124.1, 110.2, 90.9, 69.2, 57.9, 49.5, 47.1, 28.0, 25.6.

HRMS (ESI): Calculated for $C_{12}H_{13}NO_2$ $[M+H]^+$: 204.1019, found: 204.1019.

TLC (dichloromethane : methanol = 9 : 1) R*f*: 0.58 (UV, $KMnO_4$).

$[\alpha]_D^{25}$: –779.1 (c 0.3, EtOH);

Reported by the Font group [3]: $[\alpha]_D^{25}$: –441.3 (c 0.3, EtOH)

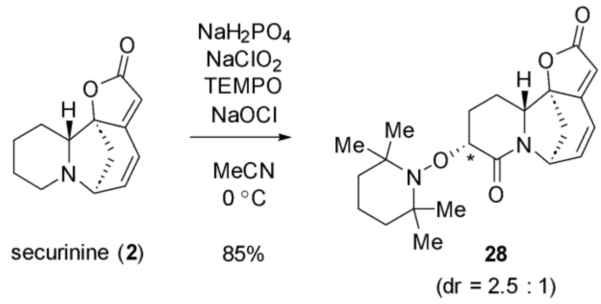

α-Alkoxyamine lactam 28:

Sodium hypochlorite solution (aq, 3%, 4.6 mL, 0.921 mmol, 1.0 equiv.) was added dropwise to a suspension of sodium phosphate monobasic anhydride (1104.4 mg, 9.205 mmol, 10.0 equiv.), sodium chlorite (249.7 mg, 2.762 mmol, 3.0 equiv.) and TEMPO (287.7 mg, 1.841 mmol, 2.0 equiv.) in acetonitrile (15.3 mL, 0.06 M) at 0 °C under argon atmosphere and stirred at 0 °C for 5 min. Then, a solution of securinine (**2**, 200 mg, 0.921 mmol, 1.0 equiv.) in acetonitrile (3.1 mL, 0.3 M) was added dropwise over 10 min to an aforementioned suspension and the resulting mixture was further stirred at 0 °C for 1 h. The resulting reaction mixture diluted with ethyl acetate (20 mL) and poured over saturated aqueous sodium thiosulfate solution (20 mL) in separatory funnel. Sodium hydroxide (aq, 3 M) was added dropwise to the mixture until pale yellow precipitates were formed. Subsequently, brine was added to redissolve the precipitate, and the aqueous layer was extracted with ethyl acetate (3 × 50 mL). Combined organic layer was dried over anhydrous sodium sulfate and concentrated under reduced pressure. The resulting crude residue was purified by flash column chromatography (silica gel: diam: 3 cm, ht: 16 cm, eluent: acetone : hexanes = 1 : 4) to afford **28** (301.3 mg, 85%, dr = 2.5 : 1) as a white powder. Analytic samples of major and minor diastereomers of **28** were isolated via iterative flash column chromatography (silica gel: diam: 3 cm, ht: 20 cm, eluent: acetone : hexanes = 1 : 4)

α-Alkoxyamine lactam 28 (major diastereomer)

^{1}H NMR (400 MHz, $CDCl_3$): δ 6.70 (dd, *J* = 9.0, 5.9 Hz, 1H), 6.63 (d, *J* = 9.4 Hz, 1H), 5.78 (s, 1H), 5.16 (t, *J* = 5.4 Hz, 1H), 4.24 (dd, *J* = 4.5, 2.6 Hz, 1H), 3.75 (dd, *J* = 11.1, 5.5 Hz, 1H), 2.41 (dd, *J* = 10.4, 5.0 Hz, 1H), 2.22 (dddd, *J* = 14.4, 8.1, 5.8, 3.2 Hz, 1H), 2.05 – 1.95 (m, 1H), 1.92 (d, *J* = 10.4 Hz, 1H), 1.90 – 1.84 (m, 1H), 1.74 (dtd, *J* = 13z.1, 10.8, 4.6 Hz, 1H), 1.52 – 1.33 (br, 5H), 1.14 – 1.08 (br, 3H), 1.04 – 0.98 (br, 4H), 0.93 – 0.88 (br, *J* = 11.1 Hz, 6H).

^{13}C NMR (101 MHz, $CDCl_3$): δ 172.1, 168.2, 167.8, 141.3, 122.3, 109.2, 89.7, 83.3, 60.9, 59.3, 55.6, 51.3, 40.0, 39.8, 33.5, 29.9, 24.8, 22.5, 21.2, 20.1, 17.3.

HRMS (ESI): Calculated for $C_{22}H_{30}N_2O_4$ $[M+H]^+$: 387.2278, found: 387.2281.

TLC (acetone : hexanes = 1 : 4) R*f*: 0.06 (UV, $KMnO_4$).

$[\alpha]_D^{25}$: –597.1 (c 0.1, EtOH)

α-Alkoxyamine lactam 28 (minor diastereomer)

^{1}H NMR (400 MHz, $CDCl_3$): δ 6.75 (dd, *J* = 9.0, 6.0 Hz, 1H), 6.60 (d, *J* = 8.9 Hz, 1H), 5.75 (s, 1H), 5.22 (t, *J* = 5.5 Hz, 1H), 4.29 (dd, *J* = 9.6, 8.0 Hz, 1H), 3.33 (dd, *J* = 11.3, 4.7 Hz, 1H), 2.47 (ddd, *J* = 15.5, 10.1, 5.6 Hz, 2H), 2.00 – 1.91 (m, 1H), 1.90 (d, *J* = 10.4 Hz, 1H), 1.86 – 1.68 (m, 2H), 1.42 – 1.37 (br, *J* = 12.8 Hz, 4H), 1.26 – 1.21 (br, *J* = 21.2 Hz, 5H), 1.14 – 1.08 (br, *J* = 10.7 Hz, 9H).

^{13}C NMR (101 MHz, $CDCl_3$): δ 171.9, 168.6, 167.7, 142.0, 122.4, 109.2, 89.5, 80.6, 61.5, 59.6, 56.1, 51.3, 40.9, 39.8, 35.1, 33.1, 25.7, 23.5, 20.7, 20.4, 17.3.

HRMS (ESI): Calculated for $C_{22}H_{30}N_2O_4$ $[M+H]^+$: 387.2278, found: 387.2285.

TLC (acetone : hexanes = 1 : 4) R*f*: 0.15 (UV, $KMnO_4$).

$[\alpha]_D^{25}$: –337.8 (c 0.1, EtOH)

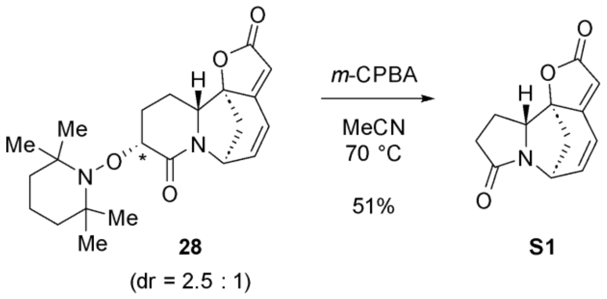

5-Oxonorsecurinine (S1):

meta-Chloroperbenzoic acid (77% purity, 496.4 mg, 2.215 mmol, 3.0 equiv.) in acetonitrile (2 mL) was added dropwise to a solution of **28** (285.4 mg, 0.738 mmol, 1.0 equiv.) in acetonitrile (53 mL, 0.014 M) at 70 °C under argon atmosphere and stirred at 70 °C. After 24 h, the resulting reaction mixture was poured over saturated aqueous sodium thiosulfate solution (30 mL) and stirred 20 min. After 20 min, saturated aqueous sodium bicarbonate solution (30 mL) was poured and extracted with ethyl acetate (3 × 60 mL). Combined organic layer was dried over anhydrous sodium sulfate and concentrated under reduced pressure. The resulting crude residue was purified by flash column chromatography (silica gel: diam: 3 cm, ht: 17 cm; eluent: ethyl acetate : hexanes = 9 : 1) to afford 5-oxonorsecurinine (**S1**, 82.3 mg, 51%) as a pale yellow powder.

^{1}H NMR (400 MHz, $CDCl_3$): δ 6.65 – 6.58 (m, 2zH), 5.77 (s, 1H), 4.91 (td, *J* = 5.0, 1.8 Hz, 1H), 3.71 (dd, *J* = 9.7, 6.6 Hz, 1H), 2.60 (ddd, *J* = 16.2, 13.5, 8.0 Hz, 1H), 2.38 – 2.18 (m, 3H), 2.07 (tdd, *J* = 13.2, 9.7, 8.5 Hz, 1H), 1.94 (d, *J* = 10.4 Hz, 1H).
^{13}C NMR (101 MHz, $CDCl_3$): δ 178.9, 172.1, 167.1, 139.9, 122.5, 109.3, 89.9, 61.0, 53.1, 41.9, 32.6, 25.9.
HRMS (ESI): Calculated for $C_{12}H_{11}NO_3$ $[M+H]^+$: 218.0812, found: 218.0815.
TLC (ethyl acetate : hexanes = 9 : 1) R*f*: 0.38 (UV, $KMnO_4$).
$[\alpha]_D^{25}$: –585.5 (c 0.1, EtOH)

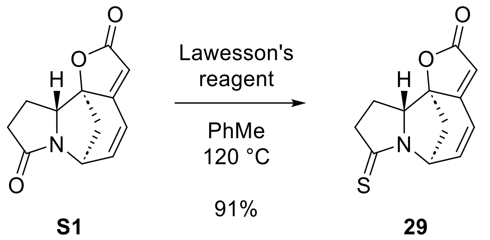

Thiolactam 29:

Lawesson's reagent (156.4 mg, 0.387 mmol, 1.2 equiv.) was added to a solution of **S1** (70.0 mg, 0.322 mmol, 1.0 equiv.) in toluene (3.2 mL, 0.1 M) at 23 °C under argon atmosphere. The resulting mixture was heated to 120 °C. After 30 min, the reaction mixture was diluted with ether (20 mL) and filtered through a pad of celite. Volatiles were evaporated under reduced pressure, and the resulting crude mixture was purified by flash column chromatography (silica gel: diam: 3 cm, ht: 14 cm; eluent: acetone : hexanes = 1 : 4) to afford thiolactam **29** (17.3 mg, 96%) as a white powder.

^{1}H NMR (400 MHz, $CDCl_3$): δ 6.73 – 6.63 (m, 2H), 5.82 (s, 1H), 5.41 (t, J = 5.3 Hz, 1H), 3.99 (dd, J = 10.5, 6.2 Hz, 1H), 3.18 – 2.88 (m, 2H), 2.34 (dd, J = 10.5, 4.7 Hz, 1H), 2.32 – 2.25 (m, 1H), 2.18 – 2.03 (m, 1H), 1.99 (d, J = 10.4 Hz, 1H).
^{13}C NMR (101 MHz, $CDCl_3$): δ 205.7, 171.7, 166.5, 138.1, 123.5, 109.8, 88.6, 66.9, 57.2, 46.2, 41.3, 28.4.
HRMS (ESI): Calculated for $C_{12}H_{11}NO_2S$ $[M+H]^+$: 234.0583, found: 234.0585.
TLC (acetone : hexanes = 1 : 4) R*f*: 0.18 (UV, $KMnO_4$).
$[\alpha]_D^{25}$: –241.3 (c 0.1, EtOH)

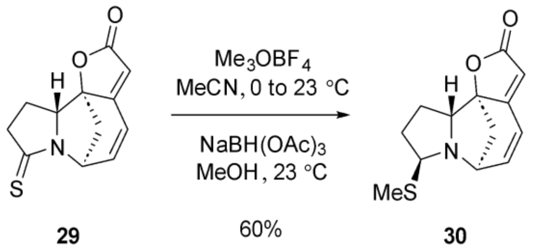

29 30

Thioaminal 30:

Trimethyloxonium tetrafluoroborate (82.0 mg, 0.554 mmol, 3.0 equiv.) was added to a solution of **29** (43.1 mg, 0.185 mmol, 1.0 equiv.) in acetonitrile (1.84 mL, 0.1 M) at 0 °C under an atmosphere of argon, stirred at 23 °C for 1 h. Volatiles were evaporated in vacuo, and the residue was dissolved in methanol (1.84 mL, 0.1 M). Sodium triacetoxyborohydride (100 mg, 0.462 mmol, 2.5 equiv.) was added to the aforementioned solution. After stirring at 23 °C for 2 h, the reaction mixture was poured to a saturated aqueous sodium bicarbonate solution (10 mL) and extracted with dichloromethane (3 × 20 mL). The combined organic layers were dried over anhydrous sodium sulfate. The filtrate was concentrated over reduced pressure, and the resulting crude residue was purified by flash column chromatography (silica gel: diam. 3 cm, ht. 13 cm; eluent: acetone : hexane = 1 : 4 to afford thioaminal **30** (27.4 mg, 60%) as a white solid.

^{1}H NMR (400 MHz, $CDCl_3$): δ 6.68 (dd, *J* = 9.0, 6.5 Hz, 1H), 6.45 (d, *J* = 9.0 Hz, 1H), 5.64 (s, 1H), 3.88 (dd, *J* = 9.8, 5.4 Hz, 1H), 3.82 (dd, *J* = 6.5, 4.7 Hz, 1H), 3.30 (t, *J* = 8.0 Hz, 1H), 2.51 (dd, *J* = 10.6, 4.7 zHz, 1H), 2.23 – 2.16 (m, 1H), 2.05 (s, 3H), 1.98 (dt, *J* = 13.4, 6.8 Hz, 1H), 1.87 (dddd, *J* = 11.1, 9.2, 6.1, 3.7 Hz, 2H), 1.75 (d, *J* = 10.6 Hz, 1H).
^{13}C NMR (101 MHz, $CDCl_3$): δ 172.9, 168.4, 144.0, 120.6, 108.1, 92.0, 71.9, 65.4, 58.5, 36.6, 33.4, 28.5, 11.0.
HRMS (ESI): Calculated for $C_{13}H_{15}NO_2S$ $[M+H]^+$: 250.0896, found: 250.0902.
TLC (ethyl acetate : hexanes = 1 : 1) R*f*: 0.15 (UV, $KMnO_4$).
$[\alpha]_D^{25}$: –147.0 (c 0.1, EtOH)

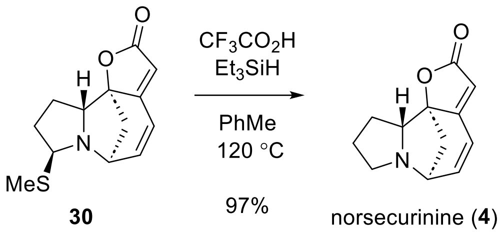

Norsecurinine (4):

Trifluoroacetic acid (0.02 mL, 0.213 mmol, 5.0 equiv.) and triethylsilane (0.03 mL, 0.213 mmol, 5.0 equiv.) was added to a solution of **30** (10.6 mg, 4.25 $\times 10^{-2}$ mmol, 1.0 equiv.) in toluene (0.43 mL, 0.1 M) at 23 °C under an atmosphere of argon. After stirring at 120 °C for 2 h, the reaction mixture was cooled down to 23 °C, and poured to a saturated aqueous sodium bicarbonate solution (5 mL) and extracted with dichloromethane (3 × 15 mL). The combined organic layers were dried over anhydrous sodium sulfate. The filtrate was concentrated over reduced pressure, and the resulting crude residue was purified by flash column chromatography (silica gel: diam. 3 cm, ht. 13 cm; eluent: dichloromethane : methanol = 10 : 1) to afford norsecurinine (**4**, 8.4 mg, 97%) as a yellow amorphous solid.

^{1}H NMR (400 MHz, $CDCl_3$): δ 6.73 (dd, *J* = 9.1, 6.4 Hz, 1H), 6.46 (d, *J* = 9.0 Hz, 1H), 5.64 (s, 1H), 3.60 (dd, *J* = 6.4, 4.7 Hz, 1H), 3.30 – 3.25 (m, 1H), 3.18 (t, *J* = 7.4 Hz, 1H), 2.54 (ddd, *J* = 19.4, 9.8, 5.2 Hz, 2H), 2.01 – 1.92 (m, 2H), 1.85 – 1.73 (m, 2H), 1.70 (d, *J* = 10.6 Hz, 1H).
^{13}C NMR (101 MHz, $CDCl_3$): δ 173.0, 168.7, 144.1, 120.6, 108.1, 92.1, 65.3, 60.0, 55.5, 36.0, 29.6, 27.0.
HRMS (ESI): Calculated for $C_{12}H_{13}NO_2$ $[M+H]^+$: 204.1019, found: 204.1020.
TLC (dichloromethane : methanol = 10 : 1) R*f*: 0.38 (UV, $KMnO_4$).
$[\alpha]_D^{25}$: –261.9 (c 0.2, EtOH);
Reported by the Tamura group [4]: $[\alpha]_D^{25}$: –272.0 (c 6.9, EtOH)

4. Spectroscopic Data Comparison Between Synthetic Natural Products with References

4.1. NMR data of previous synthetic and present synthetic allonorsecurinine (3).

Table S1. Comparison of ^{1}H NMR spectroscopic data between previous synthetic and present synthetic allonorsecurinine (3).

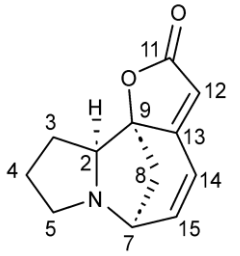

allonorsecurinine (**3**)

position	Peixoto group [5] (ppm ; multi, *J* in Hz) [a]	This work (ppm ; multi, *J* in Hz) [b]	Difference (ppm)
2	4.14 (t, J = 7.3 Hz)	4.13 (t, 7.4 Hz)	–0.01
3	1.96 – 1.57 (m, 3H)	1.63 (dtd, 12.6, 7.3, 5.1 Hz)	–0.14[c]
	1.33 – 1.17 (m)	1.23 (ddt, 12.7, 8.5, 7.4 Hz)	–0.02
4	1.96 – 1.57 (m, 3H)	1.85 (tdd, 12.2, 7.1, 5.1 Hz)	0.09[c]
	1.96 – 1.57 (m, 3H)	1.82 – 1.69 (m)	–0.01[c]
5	2.97 – 2.77 (m, 3H)	2.91 – 2.86 (m, 2H)	0.02[c]
	2.97 – 2.77 (m, 3H)	2.86 – 2.75 (m)	–0.07[c]
7	3.97 (t, J = 4.8 Hz)	3.95 (t, 4.97 Hz)	–0.02
8	2.97 – 2.77 (m, 3H)	2.91 – 2.86 (m, 2H)	0.02[c]
	2.03 (d, J = 9.9 Hz)	2.01 (d, *J* = 9.9 Hz)	–0.02
12	5.78 (s)	5.76 (s)	–0.02
14	6.70 (dd, J = 9.1, 0.9 Hz)	6.68 (dd, 9.1, 1.1 Hz)	–0.02
15	6.86 (dd, J = 9.1, 5.4 Hz)	6.84 (dd, 9.1, 5.4 Hz)	–0.02

[a] Reference: residual chloroform δ = 7.26 ppm.
[b] Reference: residual chloroform δ = 7.24 ppm.
[c] Difference in chemical shifts were calculated from median value of the chemical shift describing multiple proton signals as multiplets.

Table S2. Comparison of ^{13}C NMR spectroscopic data between previous synthetic and present synthetic allonorsecurinine (3).

position	Peixoto group [5] (ppm) [a]	This work (ppm) [b]	Difference (ppm)
2	69.2	69.2	0.0
3	25.5	25.6.	0.1
4	27.9	28.0	0.1
5	49.4	49.5	0.1
7	57.8	57.9	0.1
8	47.0	47.1	0.1
9	90.9	90.9	0.0
11	172.5	172.5	0.0
12	110.0	110.2	0.2
13	167.1	167.2	0.1
14	124.0	124.1	0.1
15	149.3	149.3	0.0

[a] Reference: $CDCl_3$ $\delta = 77.16$ ppm.
[b] Reference: $CDCl_3$ $\delta = 77.23$ ppm.

4.2. NMR data of previous synthetic and present synthetic norsecurinine (4)

Table S3. Comparison of ^{1}H NMR spectroscopic data between previous synthetic and present synthetic norsecurinine (4).

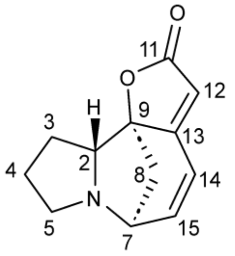

norsecurinine (**4**)

position	Peixoto group [5] (ppm ; multi, *J* in Hz) [a]	This work (ppm ; multi, *J* in Hz) [b]	Difference (ppm)
2	3.19 (dd, J = 8.5, 7.4 Hz)	3.18 (t, 7.4 Hz)	–0.01
3	2.02 – 1.93 (m, 2H)	2.01 – 1.92 (m, 2H)	–0.01[c]
	1.84 – 1.73 (m, 2H)	1.85 – 1.73 (m, 2H)	–0.01[c]
4	2.02 – 1.93 (m, 2H)	2.01 – 1.92 (m, 2H)	–0.01[c]
	1.84 – 1.73 (m, 2H)	1.85 – 1.73 (m, 2H)	–0.01[c]
5	3.28 (dd, J = 8.5, 6.8 Hz)	3.30 – 3.25 (m)	–0.01
	2.55 – 2.51 (m)	2.54 (ddd, 19.4, 9.8, 5.2 Hz, 2H)	–0.01
7	3.62 (dd, J = 6.2, 4.9 Hz)	3.60 (dd, 6.4, 4.7 Hz)	–0.02
8	2.57 (dd, J = 10.6, 4.7 Hz)	2.54 (ddd, 19.4, 9.8, 5.2 Hz, 2H)	–0.03
	1.73 – 1.70 (d, J = 10.6 Hz)	1.70 (d, *J* = 10.6 Hz)	–0.02
12	5.66 (s)	5.64 (s)	–0.02
14	6.48 (d, J = 9.0 Hz)	6.46 (d, 9.0 Hz)	–0.02
15	δ 6.74 (dd, J = 9.0, 6.4 Hz)	6.73 (dd, 9.1, 6.4 Hz)	–0.01

[a] Reference: residual chloroform δ = 7.26 ppm.
[b] Reference: residual chloroform δ = 7.24 ppm.
[c] Difference in chemical shifts were calculated from median value of the chemical shift describing multiple proton signals as multiplets.

Table S4. Comparison of ^{13}C NMR spectroscopic data between previous synthetic and present synthetic norsecurinine (4).

position	Peixoto group [5] (ppm) [a]	This work (ppm) [b]	Difference (ppm)
2	59.9	60.0	0.1
3	29.5	29.6	0.1
4	26.9	27.0	0.1
5	55.4	55.5	0.1
7	65.3	65.3	0.0
8	35.9	36.0	0.1
9	92.0	92.1	0.1
11	172.9	173.0	0.1
12	108.0	108.1	0.1
13	168.6	168.7	0.1
14	120.5	120.6	0.1
15	144.0	144.1	0.1

[a] Reference: $CDCl_3$ $\delta = 77.16$ ppm.
[b] Reference: $CDCl_3$ $\delta = 77.23$ ppm.

5. Single Crystal X-Ray Diffraction Analysis of Compounds 20 and 14

5.1. X-ray Crystal Structure of α-alkoxyamine lactam 20 (major diastereomer)

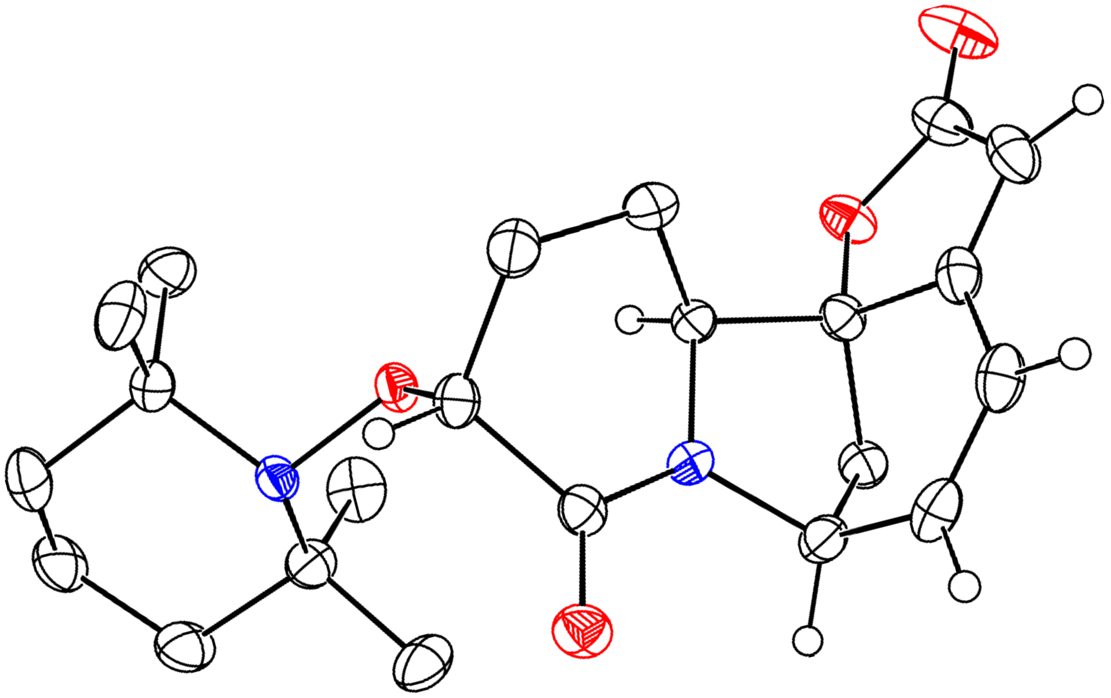

Figure S1. Thermal ellipsoid representation of α-alkoxyamine lactam **20** (major diastereomer).

Table S5. Crystal data and structure refinement for α-alkoxyamine lactam 20 (major diastereomer)

Empirical formula	$C_{22} H_{30} N_2 O_4$	
Formula weight	386.48	
Temperature	203(2) K	
Wavelength	0.71073 Å	
Crystal system	Monoclinic	
Space group	$P2_1$	
Unit cell dimensions	a = 6.2933(4) Å	α = 90°
	b = 13.8698(9) Å	β = 104.973(2)°
	c = 12.0821(7) Å	γ = 90°
Volume	1018.80(11) $Å^3$	
Z	2	
Density (calculated)	1.260 Mg/m^3	
Absorption coefficient	0.087 mm^{-1}	
F(000)	416	
Crystal size	0.288 x 0.216 x 0.114 mm^3	
Theta range for data collection	3.351 to 28.040°.	
Index ranges	–8<=h<=8, –18<=k<=18, –15<=l<=15	
Reflections collected	44854	
Independent reflections	4902 [R(int) = 0.0453]	
Completeness to theta = 25.242°	99.5 %	
Absorption correction	Semi-empirical from equivalents	
Max. and min. transmission	0.7456 and 0.7027	
Refinement method	Full-matrix least-squares on F^2	
Data / restraints / parameters	4902 / 1 / 257	
Goodness-of-fit on F^2	1.061	
Final R indices [I>2sigma(I)]	R1 = 0.0306, wR2 = 0.0791	
R indices (all data)	R1 = 0.0319, wR2 = 0.0805	
Absolute structure parameter	-0.1(2)	
Largest diff. peak and hole	0.197 and –0.145 $e·Å^{-3}$	

Table S6. Atomic coordinates (x 10^4) and equivalent isotropic displacement parameters ($Å^2$ x 10^3) for α-alkoxyamine lactam 20 (major diastereomer). U(eq) is defined as one third of the trace of the orthogonalized U^{ij} tensor.

	x	y	z	U(eq)
O(1)	9788(2)	5902(1)	9388(1)	33(1)
O(2)	11848(3)	7180(1)	10112(2)	55(1)
C(3)	10296(3)	6680(1)	10122(2)	37(1)
C(4)	8727(3)	6711(1)	10826(2)	38(1)
C(5)	7324(3)	5978(1)	10527(1)	30(1)
C(6)	5645(3)	5572(2)	11014(1)	36(1)
C(7)	4785(3)	4716(2)	10633(1)	35(1)
C(8)	5505(3)	4201(1)	9679(1)	29(1)
C(9)	7969(3)	4370(1)	9803(1)	29(1)
C(10)	7842(3)	5447(1)	9542(1)	26(1)
C(11)	6011(3)	5487(1)	8406(1)	26(1)
C(12)	4738(3)	6429(1)	8119(2)	39(1)
C(13)	2525(3)	6285(1)	7247(2)	35(1)
C(14)	2012(3)	5241(1)	6868(1)	27(1)
C(15)	2511(3)	4581(1)	7918(1)	26(1)
N(16)	4554(2)	4711(1)	8591(1)	26(1)
O(17)	1193(2)	4003(1)	8123(1)	34(1)
O(18)	3374(2)	5001(1)	6110(1)	26(1)
N(19)	2248(2)	4346(1)	5207(1)	24(1)
C(20)	1714(3)	4917(1)	4123(1)	29(1)
C(21)	626(4)	4223(2)	3157(2)	41(1)
C(22)	2004(4)	3334(2)	3108(2)	44(1)
C(23)	2491(4)	2825(1)	4259(2)	43(1)
C(24)	3647(3)	3470(1)	5273(2)	32(1)
C(25)	13(3)	5680(2)	4216(2)	41(1)
C(26)	3658(4)	5440(2)	3840(2)	40(1)
C(27)	6059(3)	3645(2)	5279(2)	46(1)
C(28)	3594(4)	2938(1)	6377(2)	45(1)

Table S7. Bond lengths [Å] and angles [°] for α-alkoxyamine lactam 20 (major diastereomer)

O(1)-C(3)	1.380(2)
O(1)-C(10)	1.4324(19)
O(2)-C(3)	1.201(2)
C(3)-C(4)	1.460(3)
C(4)-C(5)	1.334(3)
C(4)-H(4)	0.9400
C(5)-C(6)	1.448(3)
C(5)-C(10)	1.506(2)
C(6)-C(7)	1.337(3)
C(6)-H(6)	0.9400
C(7)-C(8)	1.521(2)
C(7)-H(7)	0.9400
C(8)-N(16)	1.4767(19)
C(8)-C(9)	1.537(2)
C(8)-H(8)	0.9900
C(9)-C(10)	1.524(2)
C(9)-H(9A)	0.9800
C(9)-H(9B)	0.9800
C(10)-C(11)	1.548(2)
C(11)-N(16)	1.4677(19)
C(11)-C(12)	1.525(2)
C(11)-H(11)	0.9900
C(12)-C(13)	1.527(3)
C(12)-H(12A)	0.9800
C(12)-H(12B)	0.9800
C(13)-C(14)	1.528(2)
C(13)-H(13A)	0.9800
C(13)-H(13B)	0.9800
C(14)-O(18)	1.4461(18)
C(14)-C(15)	1.530(2)
C(14)-H(14)	0.9900
C(15)-O(17)	1.223(2)
C(15)-N(16)	1.344(2)
O(18)-N(19)	1.4557(16)
N(19)-C(24)	1.490(2)

N(19)-C(20)	1.493(2)
C(20)-C(25)	1.529(2)
C(20)-C(21)	1.532(2)
C(20)-C(26)	1.534(3)
C(21)-C(22)	1.517(3)
C(21)-H(21A)	0.9800
C(21)-H(21B)	0.9800
C(22)-C(23)	1.519(3)
C(22)-H(22A)	0.9800
C(22)-H(22B)	0.9800
C(23)-C(24)	1.539(2)
C(23)-H(23A)	0.9800
C(23)-H(23B)	0.9800
C(24)-C(28)	1.532(3)
C(24)-C(27)	1.535(3)
C(25)-H(25A)	0.9700
C(25)-H(25B)	0.9700
C(25)-H(25C)	0.9700
C(26)-H(26A)	0.9700
C(26)-H(26B)	0.9700
C(26)-H(26C)	0.9700
C(27)-H(27A)	0.9700
C(27)-H(27B)	0.9700
C(27)-H(27C)	0.9700
C(28)-H(28A)	0.9700
C(28)-H(28B)	0.9700
C(28)-H(28C)	0.9700
C(3)-O(1)-C(10)	108.97(13)
O(2)-C(3)-O(1)	119.94(19)
O(2)-C(3)-C(4)	131.48(18)
O(1)-C(3)-C(4)	108.56(15)
C(5)-C(4)-C(3)	108.66(15)
C(5)-C(4)-H(4)	125.7
C(3)-C(4)-H(4)	125.7
C(4)-C(5)-C(6)	133.42(17)
C(4)-C(5)-C(10)	108.85(16)
C(6)-C(5)-C(10)	117.44(15)

C(7)-C(6)-C(5)	118.58(16)
C(7)-C(6)-H(6)	120.7
C(5)-C(6)-H(6)	120.7
C(6)-C(7)-C(8)	120.29(16)
C(6)-C(7)-H(7)	119.9
C(8)-C(7)-H(7)	119.9
N(16)-C(8)-C(7)	108.55(13)
N(16)-C(8)-C(9)	100.35(12)
C(7)-C(8)-C(9)	110.20(14)
N(16)-C(8)-H(8)	112.4
C(7)-C(8)-H(8)	112.4
C(9)-C(8)-H(8)	112.4
C(10)-C(9)-C(8)	97.56(13)
C(10)-C(9)-H(9A)	112.3
C(8)-C(9)-H(9A)	112.3
C(10)-C(9)-H(9B)	112.3
C(8)-C(9)-H(9B)	112.3
H(9A)-C(9)-H(9B)	109.9
O(1)-C(10)-C(5)	104.71(13)
O(1)-C(10)-C(9)	117.27(13)
C(5)-C(10)-C(9)	109.02(14)
O(1)-C(10)-C(11)	109.56(13)
C(5)-C(10)-C(11)	114.83(13)
C(9)-C(10)-C(11)	101.89(12)
N(16)-C(11)-C(12)	110.50(14)
N(16)-C(11)-C(10)	100.93(12)
C(12)-C(11)-C(10)	117.33(13)
N(16)-C(11)-H(11)	109.2
C(12)-C(11)-H(11)	109.2
C(10)-C(11)-H(11)	109.2
C(11)-C(12)-C(13)	112.13(15)
C(11)-C(12)-H(12A)	109.2
C(13)-C(12)-H(12A)	109.2
C(11)-C(12)-H(12B)	109.2
C(13)-C(12)-H(12B)	109.2
H(12A)-C(12)-H(12B)	107.9
C(12)-C(13)-C(14)	114.52(14)
C(12)-C(13)-H(13A)	108.6

C(14)-C(13)-H(13A)	108.6
C(12)-C(13)-H(13B)	108.6
C(14)-C(13)-H(13B)	108.6
H(13A)-C(13)-H(13B)	107.6
O(18)-C(14)-C(13)	107.05(13)
O(18)-C(14)-C(15)	111.10(12)
C(13)-C(14)-C(15)	109.69(13)
O(18)-C(14)-H(14)	109.7
C(13)-C(14)-H(14)	109.7
C(15)-C(14)-H(14)	109.7
O(17)-C(15)-N(16)	123.96(15)
O(17)-C(15)-C(14)	123.68(14)
N(16)-C(15)-C(14)	112.36(14)
C(15)-N(16)-C(11)	122.96(13)
C(15)-N(16)-C(8)	125.31(13)
C(11)-N(16)-C(8)	111.21(12)
C(14)-O(18)-N(19)	111.39(11)
O(18)-N(19)-C(24)	107.59(12)
O(18)-N(19)-C(20)	106.33(11)
C(24)-N(19)-C(20)	118.39(13)
N(19)-C(20)-C(25)	107.80(13)
N(19)-C(20)-C(21)	106.83(14)
C(25)-C(20)-C(21)	107.89(15)
N(19)-C(20)-C(26)	115.75(14)
C(25)-C(20)-C(26)	107.66(16)
C(21)-C(20)-C(26)	110.64(15)
C(22)-C(21)-C(20)	113.16(16)
C(22)-C(21)-H(21A)	108.9
C(20)-C(21)-H(21A)	108.9
C(22)-C(21)-H(21B)	108.9
C(20)-C(21)-H(21B)	108.9
H(21A)-C(21)-H(21B)	107.8
C(21)-C(22)-C(23)	108.90(16)
C(21)-C(22)-H(22A)	109.9
C(23)-C(22)-H(22A)	109.9
C(21)-C(22)-H(22B)	109.9
C(23)-C(22)-H(22B)	109.9
H(22A)-C(22)-H(22B)	108.3

C(22)-C(23)-C(24)	113.64(15)
C(22)-C(23)-H(23A)	108.8
C(24)-C(23)-H(23A)	108.8
C(22)-C(23)-H(23B)	108.8
C(24)-C(23)-H(23B)	108.8
H(23A)-C(23)-H(23B)	107.7
N(19)-C(24)-C(28)	107.06(14)
N(19)-C(24)-C(27)	116.21(15)
C(28)-C(24)-C(27)	108.43(17)
N(19)-C(24)-C(23)	106.24(15)
C(28)-C(24)-C(23)	107.50(16)
C(27)-C(24)-C(23)	111.01(16)
C(20)-C(25)-H(25A)	109.5
C(20)-C(25)-H(25B)	109.5
H(25A)-C(25)-H(25B)	109.5
C(20)-C(25)-H(25C)	109.5
H(25A)-C(25)-H(25C)	109.5
H(25B)-C(25)-H(25C)	109.5
C(20)-C(26)-H(26A)	109.5
C(20)-C(26)-H(26B)	109.5
H(26A)-C(26)-H(26B)	109.5
C(20)-C(26)-H(26C)	109.5
H(26A)-C(26)-H(26C)	109.5
H(26B)-C(26)-H(26C)	109.5
C(24)-C(27)-H(27A)	109.5
C(24)-C(27)-H(27B)	109.5
H(27A)-C(27)-H(27B)	109.5
C(24)-C(27)-H(27C)	109.5
H(27A)-C(27)-H(27C)	109.5
H(27B)-C(27)-H(27C)	109.5
C(24)-C(28)-H(28A)	109.5
C(24)-C(28)-H(28B)	109.5
H(28A)-C(28)-H(28B)	109.5
C(24)-C(28)-H(28C)	109.5
H(28A)-C(28)-H(28C)	109.5
H(28B)-C(28)-H(28C)	109.5

Symmetry transformations used to generate equivalent atoms:

Table S8. Anisotropic displacement parameters ($Å^2 \times 10^3$) for α-alkoxyamine lactam 20 (major diastereomer). The anisotropic displacement factor exponent takes the form: $-2\pi^2[h^2a^{*2}U^{11} + ... + 2 h k a^* b^* U^{12}]$

	U^{11}	U^{22}	U^{33}	U^{23}	U^{13}	U^{12}
O(1)	29(1)	31(1)	43(1)	-9(1)	14(1)	-6(1)
O(2)	44(1)	36(1)	87(1)	-18(1)	20(1)	-13(1)
C(3)	33(1)	27(1)	50(1)	-7(1)	6(1)	1(1)
C(4)	40(1)	34(1)	37(1)	-11(1)	4(1)	5(1)
C(5)	32(1)	33(1)	24(1)	-3(1)	5(1)	8(1)
C(6)	37(1)	48(1)	24(1)	-1(1)	12(1)	8(1)
C(7)	34(1)	47(1)	25(1)	9(1)	11(1)	4(1)
C(8)	31(1)	29(1)	27(1)	6(1)	7(1)	0(1)
C(9)	29(1)	27(1)	30(1)	0(1)	7(1)	3(1)
C(10)	26(1)	27(1)	26(1)	-3(1)	8(1)	0(1)
C(11)	32(1)	25(1)	23(1)	0(1)	9(1)	-5(1)
C(12)	48(1)	24(1)	38(1)	5(1)	1(1)	-1(1)
C(13)	43(1)	31(1)	29(1)	-1(1)	6(1)	9(1)
C(14)	27(1)	32(1)	23(1)	-1(1)	7(1)	2(1)
C(15)	28(1)	28(1)	25(1)	-3(1)	10(1)	0(1)
N(16)	30(1)	25(1)	24(1)	3(1)	7(1)	-3(1)
O(17)	32(1)	36(1)	36(1)	-1(1)	14(1)	-7(1)
O(18)	26(1)	30(1)	21(1)	-4(1)	6(1)	-4(1)
N(19)	26(1)	25(1)	22(1)	-3(1)	5(1)	-3(1)
C(20)	33(1)	32(1)	22(1)	1(1)	6(1)	0(1)
C(21)	44(1)	50(1)	25(1)	-8(1)	2(1)	-4(1)
C(22)	58(1)	42(1)	32(1)	-14(1)	13(1)	-9(1)
C(23)	61(1)	30(1)	41(1)	-9(1)	19(1)	-6(1)
C(24)	40(1)	28(1)	30(1)	-2(1)	12(1)	3(1)
C(25)	43(1)	46(1)	29(1)	6(1)	3(1)	15(1)
C(26)	49(1)	39(1)	35(1)	4(1)	18(1)	-8(1)
C(27)	35(1)	54(1)	50(1)	-4(1)	13(1)	10(1)
C(28)	71(1)	29(1)	37(1)	4(1)	19(1)	12(1)

Table S9. Hydrogen coordinates (x 10^4) and isotropic displacement parameters ($Å^2$ x 10^3) for α-alkoxyamine lactam 20 (major diastereomer).

	x	y	z	U(eq)
H(4)	8710	7171	11395	45
H(6)	5171	5904	11585	43
H(7)	3731	4430	10957	42
H(8)	5120	3507	9635	35
H(9A)	8864	4230	10578	35
H(9B)	8516	4000	9242	35
H(11)	6641	5308	7763	31
H(12A)	5620	6889	7812	46
H(12B)	4491	6704	8822	46
H(13A)	2499	6677	6569	42
H(13B)	1359	6523	7578	42
H(14)	441	5182	6452	32
H(21A)	-800	4021	3263	49
H(21B)	355	4564	2423	49
H(22A)	3382	3521	2932	52
H(22B)	1204	2900	2503	52
H(23A)	3422	2263	4235	51
H(23B)	1106	2591	4389	51
H(25A)	-563	5982	3475	61
H(25B)	703	6164	4772	61
H(25C)	-1179	5376	4459	61
H(26A)	3109	5912	3243	59
H(26B)	4572	4975	3578	59
H(26C)	4522	5762	4521	59
H(27A)	6888	3053	5485	69
H(27B)	6674	4143	5832	69
H(27C)	6138	3849	4522	69
H(28A)	4060	2276	6333	67
H(28B)	2109	2948	6471	67
H(28C)	4579	3254	7027	67

5.2. X-ray Crystal Structure of 5-oxoallonorsecurinine (14)

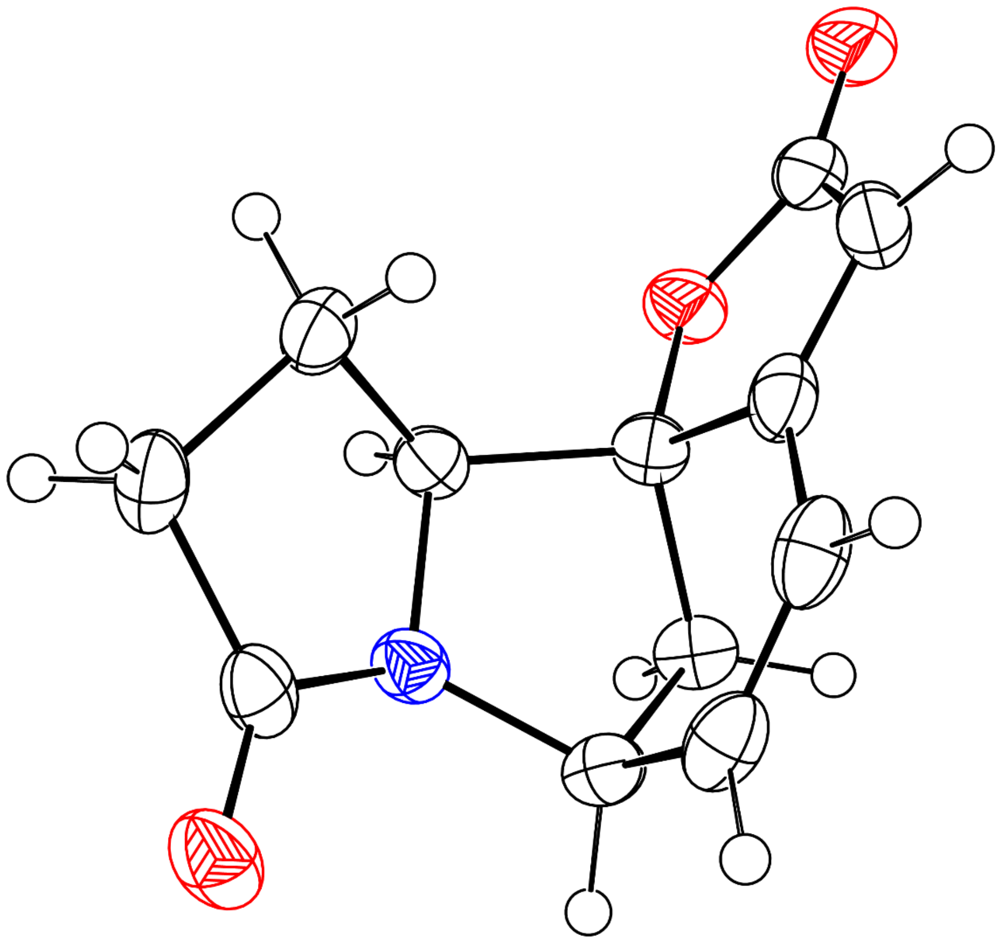

Figure S2. Thermal ellipsoid representation of 5-oxoallonorsecurinine (**14**).

Table S10. Crystal data and structure refinement for 5-oxoallonorsecurinine (14)

Empirical formula	$C_{12} H_{11} N O_3$	
Formula weight	217.22	
Temperature	173(2) K	
Wavelength	0.71073 Å	
Crystal system	Monoclinic	
Space group	$P2_1$	
Unit cell dimensions	a = 6.1801(3) Å	α = 90°
	b = 12.7128(6) Å	β = 113.5828(14)°
	c = 6.8828(3) Å	γ = 90°
Volume	495.59(4) $Å^3$	
Z	2	
Density (calculated)	1.456 Mg/m^3	
Absorption coefficient	0.106 mm^{-1}	
F(000)	228	
Crystal size	0.183 x 0.157 x 0.041 mm^3	
Theta range for data collection	3.205 to 27.007°.	
Index ranges	–7<=h<=7, –16<=k<=16, –8<=l<=8	
Reflections collected	25905	
Independent reflections	2163 [R(int) = 0.0383]	
Completeness to theta = 25.242°	100.0 %	
Absorption correction	Semi-empirical from equivalents	
Max. and min. transmission	0.7455 and 0.7057	
Refinement method	Full-matrix least-squares on F^2	
Data / restraints / parameters	2163 / 1 / 145	
Goodness-of-fit on F^2	1.057	
Final R indices [I>2sigma(I)]	R1 = 0.0276, wR2 = 0.0728	
R indices (all data)	R1 = 0.0281, wR2 = 0.0731	
Absolute structure parameter	-0.1(2)	
Largest diff. peak and hole	0.228 and –0.139 e·$Å^{-3}$	

Table S11. Atomic coordinates (x 10^4) and equivalent isotropic displacement parameters ($Å^2$ x 10^3) for 5-oxoallonorsecurinine (14). U(eq) is defined as one third of the trace of the orthogonalized U^{ij} tensor.

	x	y	z	U(eq)
O(1)	9284(3)	3341(1)	4307(3)	39(1)
C(2)	8363(4)	3501(1)	5553(3)	31(1)
C(3)	9367(4)	3277(2)	7911(4)	35(1)
C(4)	8012(4)	4034(2)	8768(3)	33(1)
C(5)	5628(3)	4118(1)	6884(3)	27(1)
N(6)	6193(3)	3933(1)	5031(2)	28(1)
C(7)	5084(3)	4739(2)	3379(3)	30(1)
C(8)	6741(3)	5672(2)	3809(3)	34(1)
C(9)	7044(3)	6322(2)	5433(3)	35(1)
C(10)	5694(3)	6135(2)	6700(3)	31(1)
C(11)	4231(3)	5156(2)	6270(3)	27(1)
C(12)	2938(3)	5036(2)	3858(3)	30(1)
O(13)	2781(3)	5280(1)	7440(2)	34(1)
O(14)	2373(3)	6453(2)	9689(3)	53(1)
C(15)	3363(4)	6202(2)	8566(3)	38(1)
C(16)	5201(4)	6719(2)	8090(3)	40(1)

Table S12. Bond lengths [Å] and angles [°] for 5-oxoallonorsecurinine (14).

O(1)-C(2)	1.220(2)
C(2)-N(6)	1.358(2)
C(2)-C(3)	1.515(3)
C(3)-C(4)	1.540(3)
C(3)-H(3A)	0.9900
C(3)-H(3B)	0.9900
C(4)-C(5)	1.528(3)
C(4)-H(4A)	0.9900
C(4)-H(4B)	0.9900
C(5)-N(6)	1.469(2)
C(5)-C(11)	1.540(3)
C(5)-H(5)	1.0000
N(6)-C(7)	1.480(2)
C(7)-C(8)	1.517(3)
C(7)-C(12)	1.536(2)
C(7)-H(7)	1.0000
C(8)-C(9)	1.341(3)
C(8)-H(8)	0.9500
C(9)-C(10)	1.447(3)
C(9)-H(9)	0.9500
C(10)-C(16)	1.339(3)
C(10)-C(11)	1.497(3)
C(11)-O(13)	1.434(2)
C(11)-C(12)	1.535(3)
C(12)-H(12A)	0.9900
C(12)-H(12B)	0.9900
O(13)-C(15)	1.372(3)
O(14)-C(15)	1.205(3)
C(15)-C(16)	1.458(4)
C(16)-H(16)	0.9500
O(1)-C(2)-N(6)	124.77(19)
O(1)-C(2)-C(3)	127.96(19)
N(6)-C(2)-C(3)	107.28(16)
C(2)-C(3)-C(4)	103.92(16)
C(2)-C(3)-H(3A)	111.0

C(4)-C(3)-H(3A)	111.0
C(2)-C(3)-H(3B)	111.0
C(4)-C(3)-H(3B)	111.0
H(3A)-C(3)-H(3B)	109.0
C(5)-C(4)-C(3)	101.87(16)
C(5)-C(4)-H(4A)	111.4
C(3)-C(4)-H(4A)	111.4
C(5)-C(4)-H(4B)	111.4
C(3)-C(4)-H(4B)	111.4
H(4A)-C(4)-H(4B)	109.3
N(6)-C(5)-C(4)	104.04(15)
N(6)-C(5)-C(11)	101.60(14)
C(4)-C(5)-C(11)	122.46(16)
N(6)-C(5)-H(5)	109.3
C(4)-C(5)-H(5)	109.3
C(11)-C(5)-H(5)	109.3
C(2)-N(6)-C(5)	112.82(15)
C(2)-N(6)-C(7)	126.01(16)
C(5)-N(6)-C(7)	110.97(15)
N(6)-C(7)-C(8)	108.84(15)
N(6)-C(7)-C(12)	100.36(14)
C(8)-C(7)-C(12)	109.82(16)
N(6)-C(7)-H(7)	112.4
C(8)-C(7)-H(7)	112.4
C(12)-C(7)-H(7)	112.4
C(9)-C(8)-C(7)	120.17(17)
C(9)-C(8)-H(8)	119.9
C(7)-C(8)-H(8)	119.9
C(8)-C(9)-C(10)	118.61(18)
C(8)-C(9)-H(9)	120.7
C(10)-C(9)-H(9)	120.7
C(16)-C(10)-C(9)	133.7(2)
C(16)-C(10)-C(11)	108.03(19)
C(9)-C(10)-C(11)	117.75(17)
O(13)-C(11)-C(10)	105.16(16)
O(13)-C(11)-C(12)	116.50(15)
C(10)-C(11)-C(12)	107.92(15)
O(13)-C(11)-C(5)	110.38(15)

C(10)-C(11)-C(5)	115.49(15)
C(12)-C(11)-C(5)	101.79(15)
C(11)-C(12)-C(7)	97.79(14)
C(11)-C(12)-H(12A)	112.2
C(7)-C(12)-H(12A)	112.2
C(11)-C(12)-H(12B)	112.2
C(7)-C(12)-H(12B)	112.2
H(12A)-C(12)-H(12B)	109.8
C(15)-O(13)-C(11)	109.24(16)
O(14)-C(15)-O(13)	120.2(2)
O(14)-C(15)-C(16)	131.7(2)
O(13)-C(15)-C(16)	108.07(17)
C(10)-C(16)-C(15)	109.4(2)
C(10)-C(16)-H(16)	125.3
C(15)-C(16)-H(16)	125.3

Symmetry transformations used to generate equivalent atoms:

Table S13. Anisotropic displacement parameters ($Å^2 \times 10^3$) for 5-oxoallonorsecurinine (14). The anisotropic displacement factor exponent takes the form: $-2\pi^2[h^2a^{*2}U^{11} + ... + 2 h k a^* b^* U^{12}]$

	U^{11}	U^{22}	U^{33}	U^{23}	U^{13}	U^{12}
O(1)	43(1)	34(1)	53(1)	-1(1)	32(1)	2(1)
C(2)	36(1)	21(1)	42(1)	1(1)	21(1)	-2(1)
C(3)	37(1)	29(1)	45(1)	12(1)	21(1)	7(1)
C(4)	37(1)	30(1)	32(1)	7(1)	14(1)	4(1)
C(5)	31(1)	24(1)	30(1)	-1(1)	18(1)	-2(1)
N(6)	31(1)	27(1)	30(1)	-1(1)	17(1)	-1(1)
C(7)	29(1)	35(1)	25(1)	1(1)	12(1)	1(1)
C(8)	26(1)	38(1)	38(1)	13(1)	15(1)	2(1)
C(9)	25(1)	29(1)	44(1)	11(1)	7(1)	-2(1)
C(10)	29(1)	25(1)	30(1)	5(1)	3(1)	3(1)
C(11)	25(1)	30(1)	28(1)	-1(1)	13(1)	-1(1)
C(12)	25(1)	37(1)	28(1)	-1(1)	11(1)	-3(1)
O(13)	36(1)	40(1)	34(1)	-2(1)	20(1)	4(1)
O(14)	74(1)	56(1)	35(1)	3(1)	27(1)	28(1)
C(15)	46(1)	37(1)	26(1)	3(1)	10(1)	17(1)
C(16)	48(1)	27(1)	33(1)	-2(1)	3(1)	6(1)

Table S14. Hydrogen coordinates (x 10^4) and isotropic displacement parameters ($Å^2$ x 10^3) for 5-oxoallonorsecurinine (14).

	x	y	z	U(eq)
H(3A)	11084	3421	8565	42
H(3B)	9090	2536	8189	42
H(4A)	7838	3738	10029	39
H(4B)	8804	4727	9135	39
H(5)	4564	3546	6978	32
H(7)	4617	4453	1914	35
H(8)	7566	5797	2926	40
H(9)	8122	6893	5745	42
H(12A)	2199	5703	3164	36
H(12B)	1738	4470	3463	36
H(16)	5934	7368	8676	48

6. Supplemental References

1. Still, W. C.; Kahn, M.; Mitra, A. Rapid chromatographic technique for preparative separations with moderate resolution. *J. Org. Chem.* **1978**, *43*, 2923–2925.
2. Kang, G.; Han, S. Synthesis of Dimeric Securinega Alkaloid Flueggeacosine B: From Pd-Catalyzed Cross-Coupling to Cu-Catalyzed Cross-Dehydrogenative Coupling. *J. Am. Chem. Soc.* **2022**, *144*, 8932–8937.
3. Alibés, R.; Ballbé, M.; Busqué, F.; de March, P.; Elias, L.; Figueredo, M.; Font, J. A New General Access to Either Type of *Securinega* Alkaloids: Synthesis of Securinine and (–)-Allonorsecurinine. *Organic Letters*. **2014**, *6*, 1813–1816.
4. Saito, S.; T. Tanaka.; K. Kotera.; H. Nakai.; N. Sugimoto.; Z. Horii.; M. Ikeda.; Y. Tamura. Structures of Norsecurinine and Dihydronorsecurinine. *Chemical & Pharmaceutical Bulletin.* **1965**, *13*, 786–796.
5. Antien, K.; Lacambra, A.; Cossío, F. P.; Massip, S.; Deffieux, D.; Pouységu, L.; Peixoto, P. A.; Quideau, S. Bio-Inspired Total Synthesis of Twelve Securinega Alkaloids: Structural Reassignments of (+)-Virosine B and (–)-Episecurinol A. *Chem. Eur. J.* **2019**, *25,* 11574–11580.

7. Copies of NMR spectra of newly Synthesized Compounds

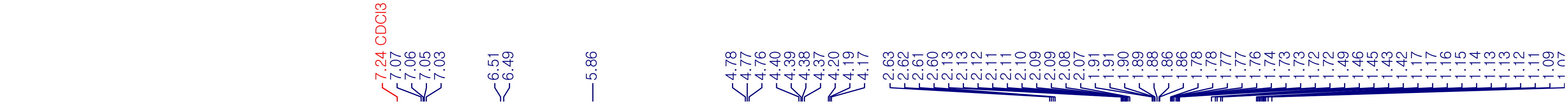

Parameter	Value
1 Origin	Bruker BioSpin GmbH
2 Solvent	CDCl3
3 Temperature	298.0
4 Experiment	1D
5 Probe	Z116098_0402 (PA BBO 400S1 BBF-H-D-05 Z SP)
6 Number of Scans	16
7 Acquisition Time	4.0894
8 Spectrometer Frequency	400.12
9 Nucleus	1H

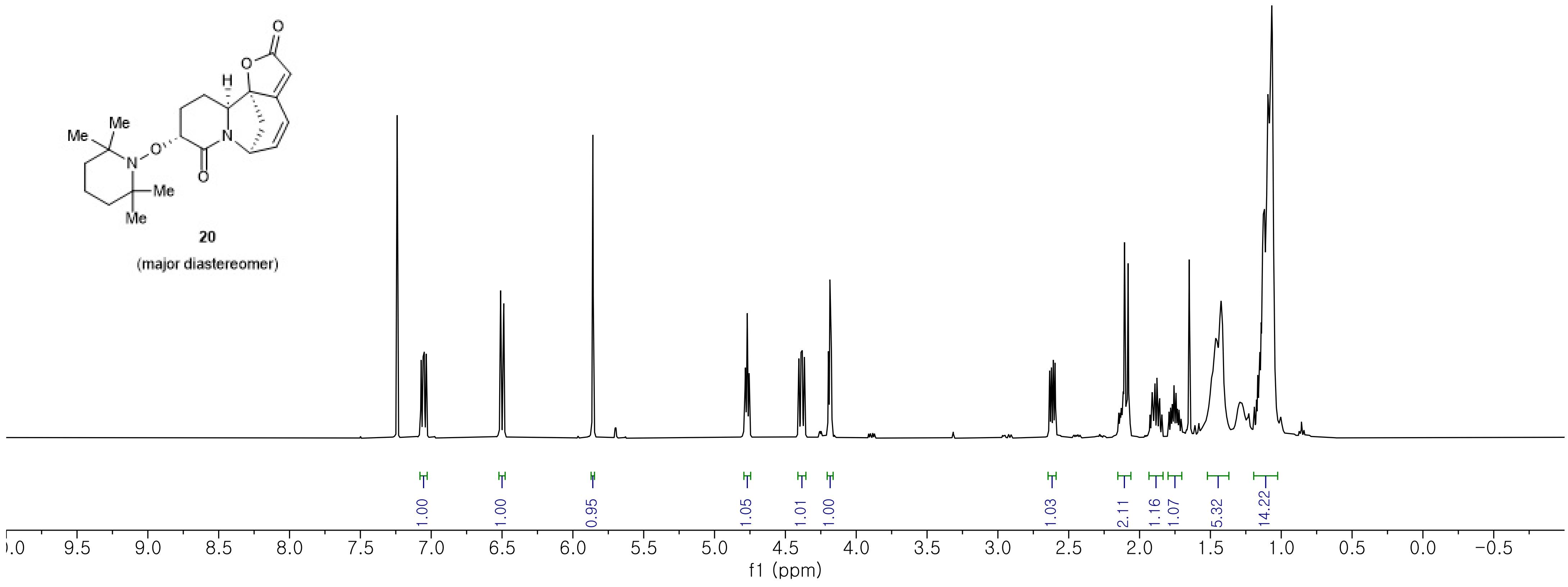

Parameter	Value
1 Origin	Bruker BioSpin GmbH
2 Solvent	CDCl3
3 Temperature	298.0
4 Experiment	1D
5 Probe	Z116098_0402 (PA BBO 400S1 BBF-H-D-05 Z SP)
6 Number of Scans	1024
7 Acquisition Time	1.3631
8 Spectrometer Frequency	100.62
9 Nucleus	13C

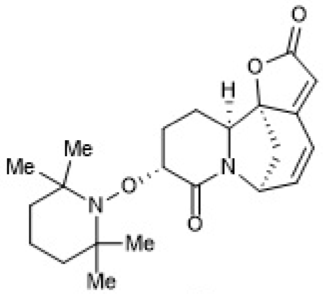

20
(major diastereomer)

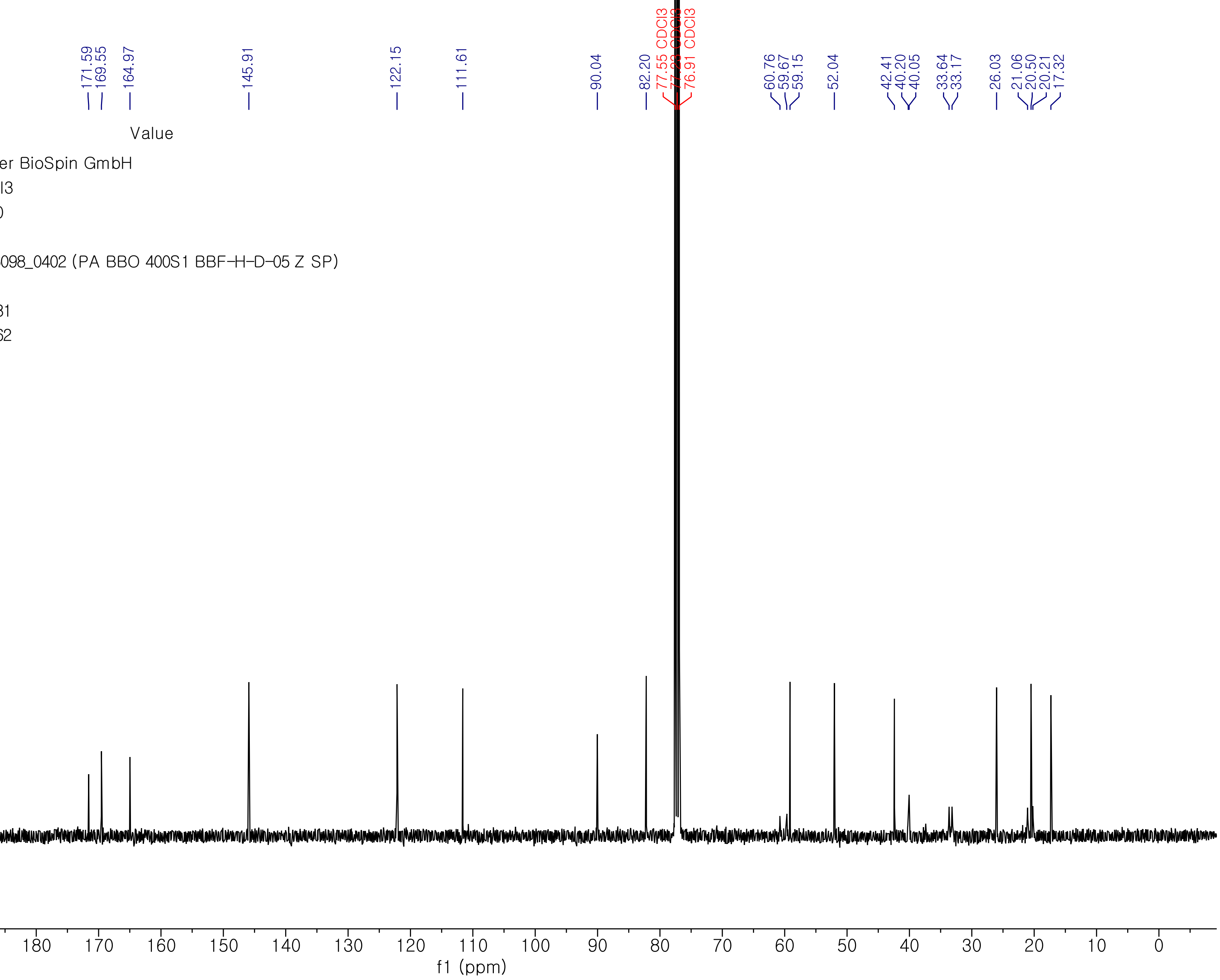

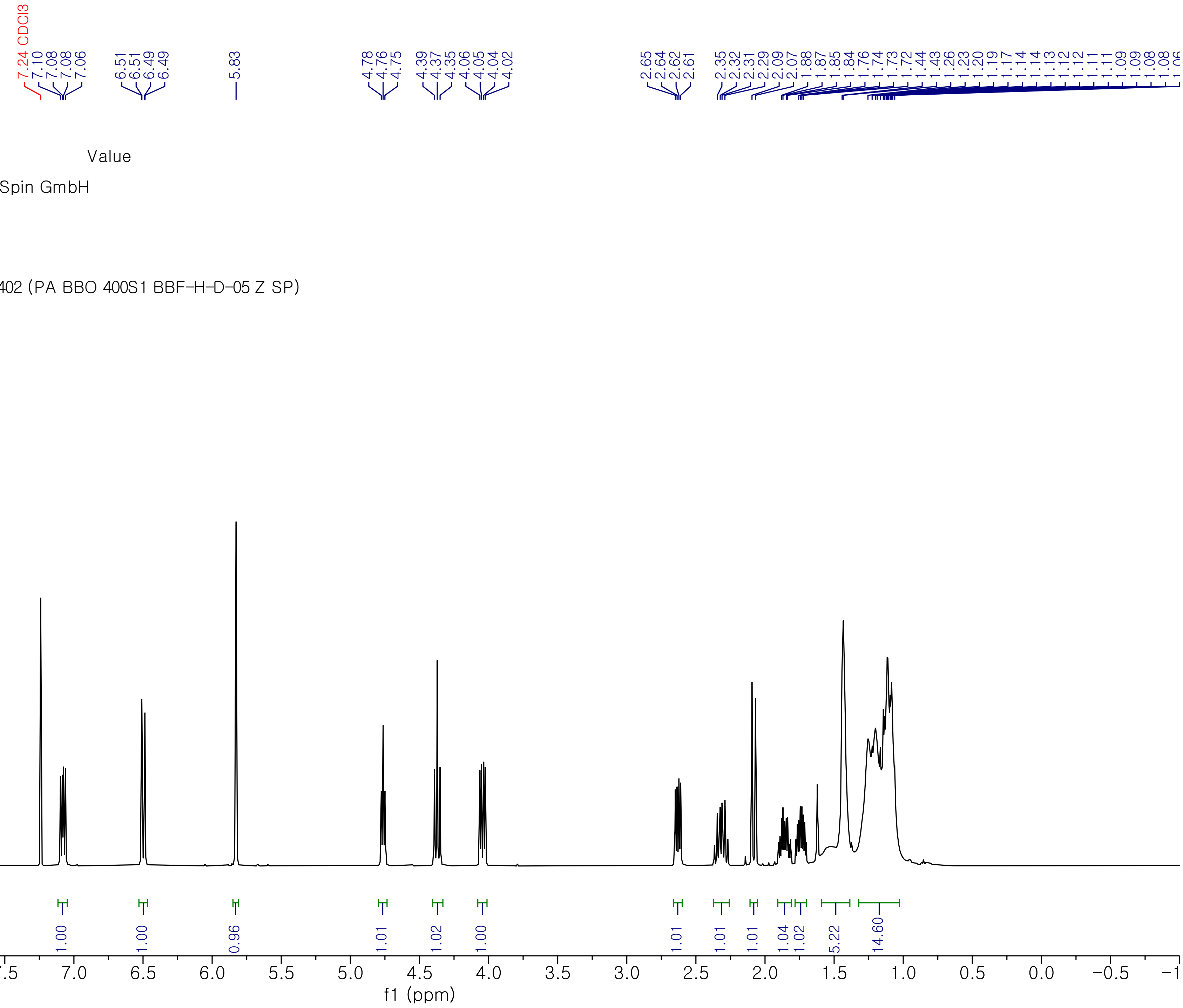

Parameter	Value
1 Origin	Bruker BioSpin GmbH
2 Solvent	CDCl3
3 Temperature	298.0
4 Experiment	1D
5 Probe	Z116098_0402 (PA BBO 400S1 BBF-H-D-05 Z SP)
6 Number of Scans	16
7 Acquisition Time	4.0894
8 Spectrometer Frequency	400.12
9 Nucleus	1H

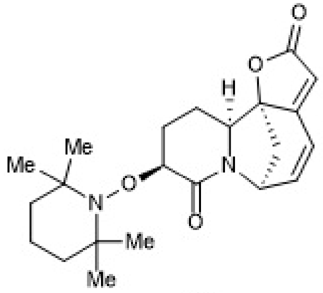

20

(minor diastereomer)

Parameter	Value
1 Origin	Bruker BioSpin GmbH
2 Solvent	CDCl3
3 Temperature	298.0
4 Experiment	1D
5 Probe	Z116098_0402 (PA BBO 400S1 BBF-H-D-05 Z SP)
6 Number of Scans	1024
7 Acquisition Time	1.3631
8 Spectrometer Frequency	100.62
9 Nucleus	13C

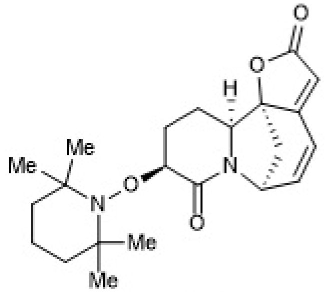

20
(minor diastereomer)

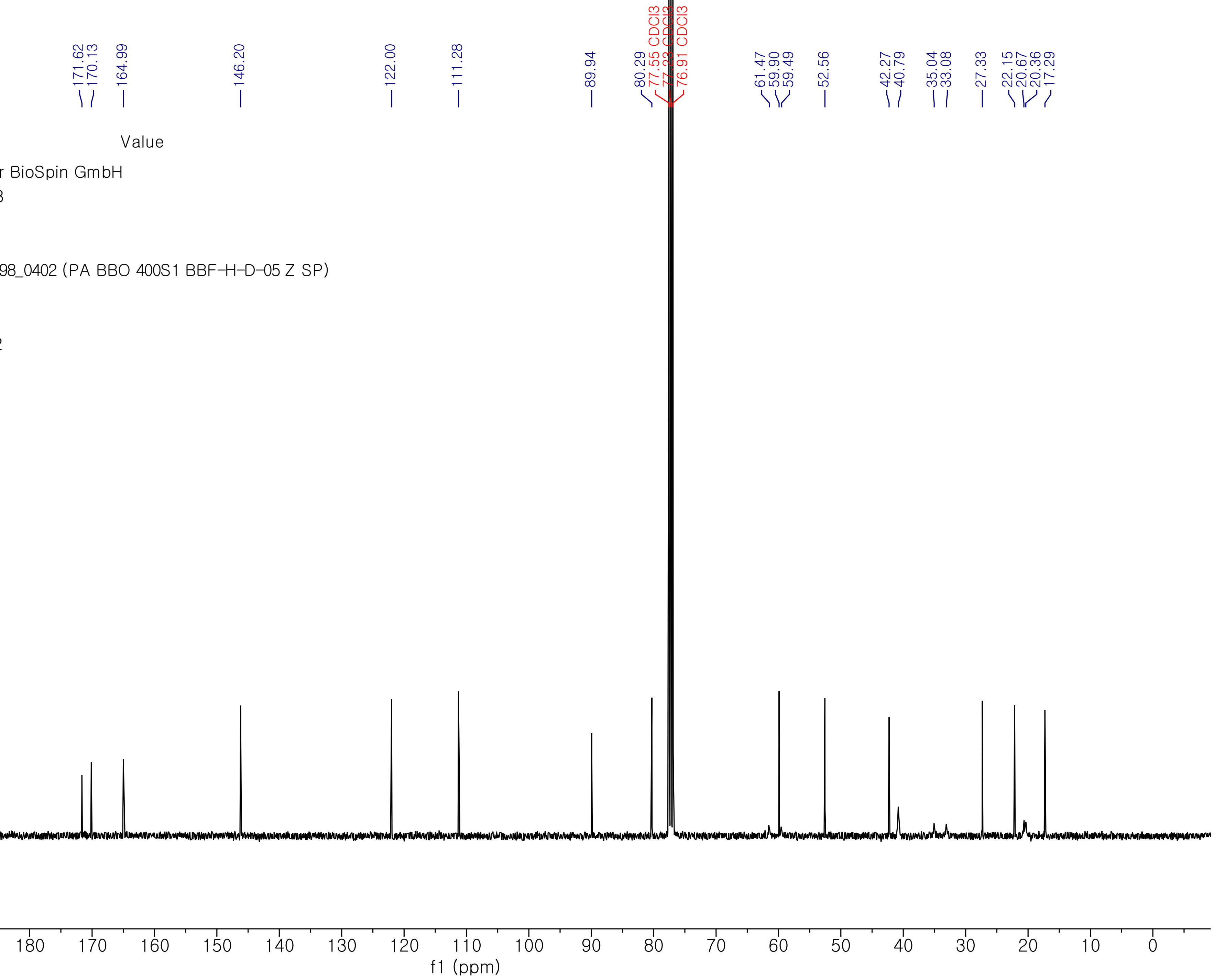

Parameter	Value
1 Origin	Bruker BioSpin GmbH
2 Solvent	CDCl3
3 Temperature	298.0
4 Experiment	1D
5 Probe	Z116098_0402 (PA BBO 400S1 BBF-H-D-05 Z SP)
6 Number of Scans	16
7 Acquisition Time	4.0894
8 Spectrometer Frequency	400.12
9 Nucleus	1H

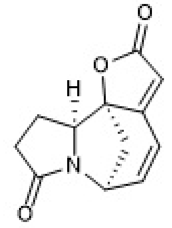

14

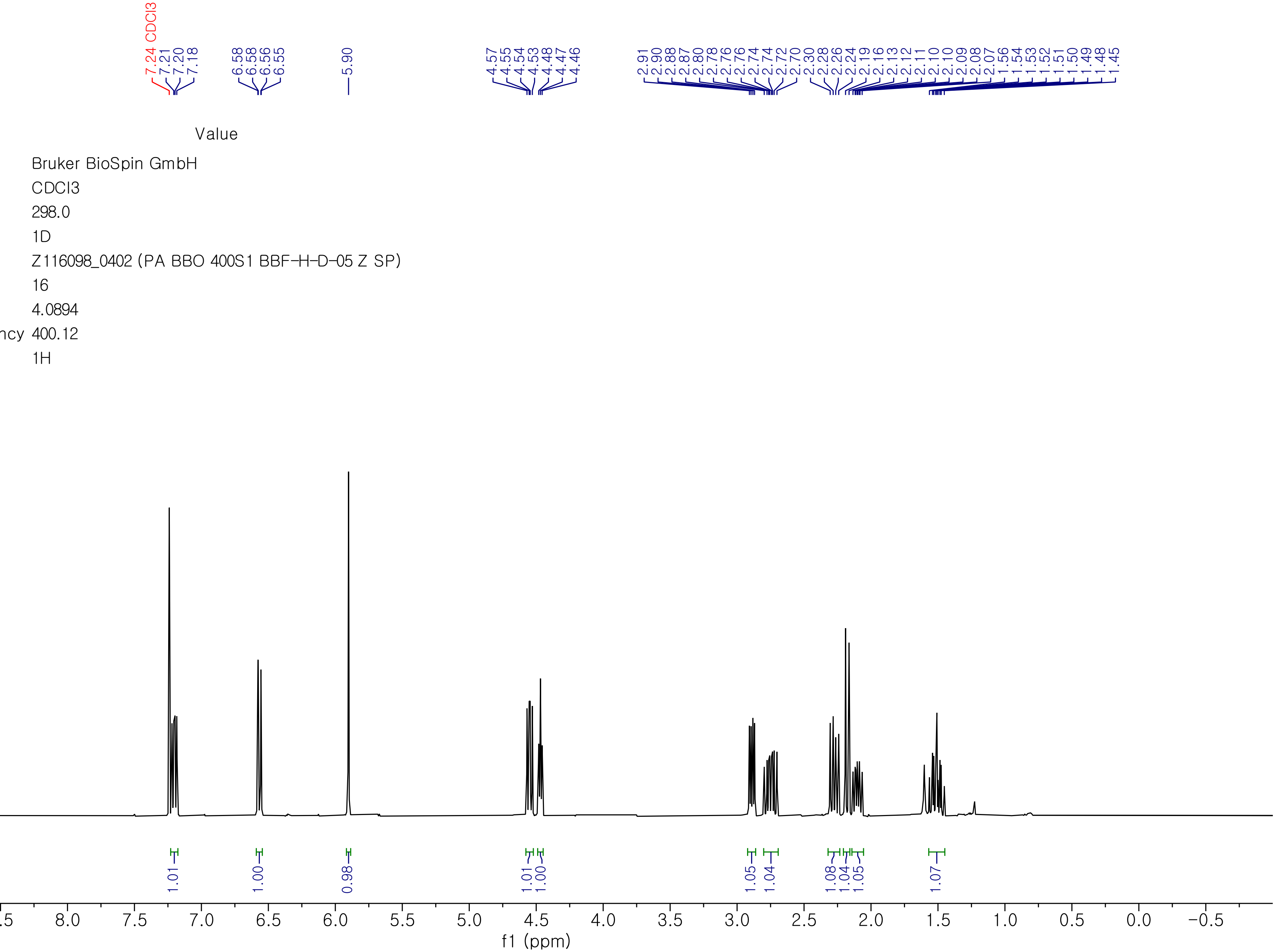

Parameter	Value
1 Origin	Bruker BioSpin GmbH
2 Solvent	CDCl3
3 Temperature	298.0
4 Experiment	1D
5 Probe	Z116098_0402 (PA BBO 400S1 BBF-H-D-05 Z SP)
6 Number of Scans	2048
7 Acquisition Time	1.3631
8 Spectrometer Frequency	100.62
9 Nucleus	13C

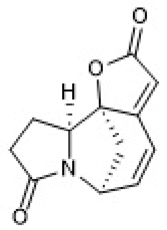

14

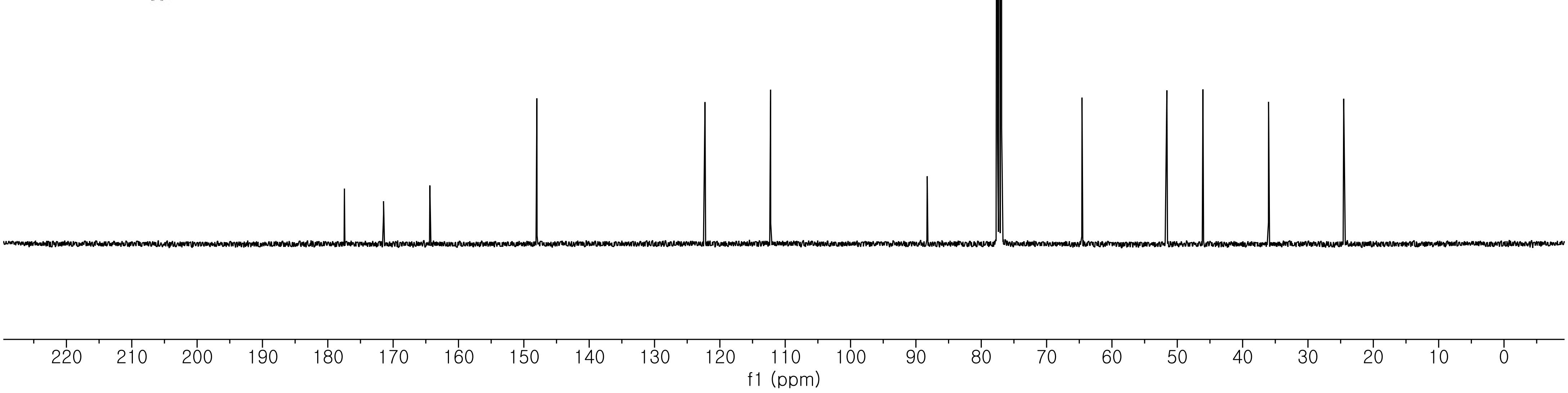

Parameter	Value
1 Origin	Bruker BioSpin GmbH
2 Solvent	CDCl3
3 Temperature	298.0
4 Experiment	1D
5 Probe	Z116098_0402 (PA BBO 400S1 BBF-H-D-05 Z SP)
6 Number of Scans	16
7 Acquisition Time	4.0894
8 Spectrometer Frequency	400.12
9 Nucleus	1H

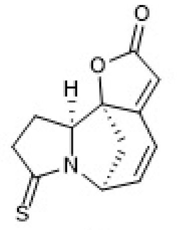

21

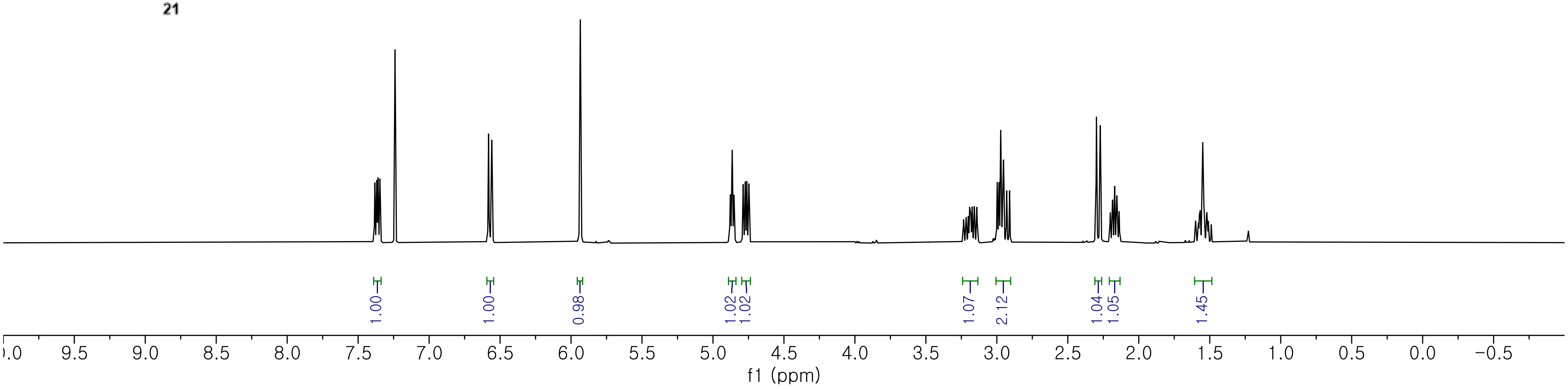

Parameter	Value
1 Origin	Bruker BioSpin GmbH
2 Solvent	CDCl3
3 Temperature	298.0
4 Experiment	1D
5 Probe	Z116098_0402 (PA BBO 400S1 BBF-H-D-05 Z SP)
6 Number of Scans	2048
7 Acquisition Time	1.3631
8 Spectrometer Frequency	100.62
9 Nucleus	13C

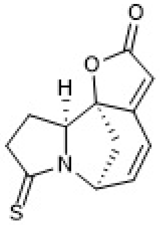

21

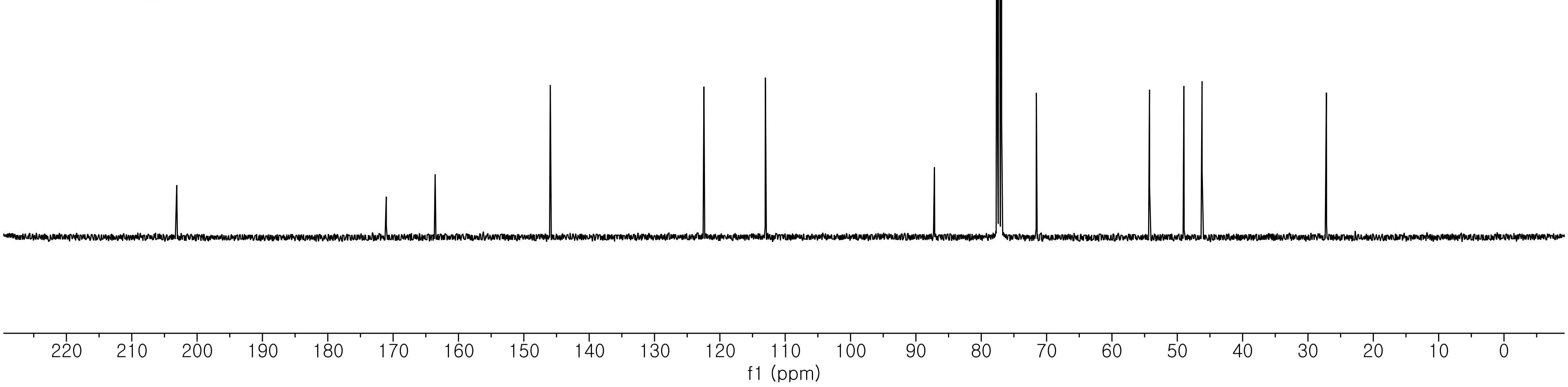

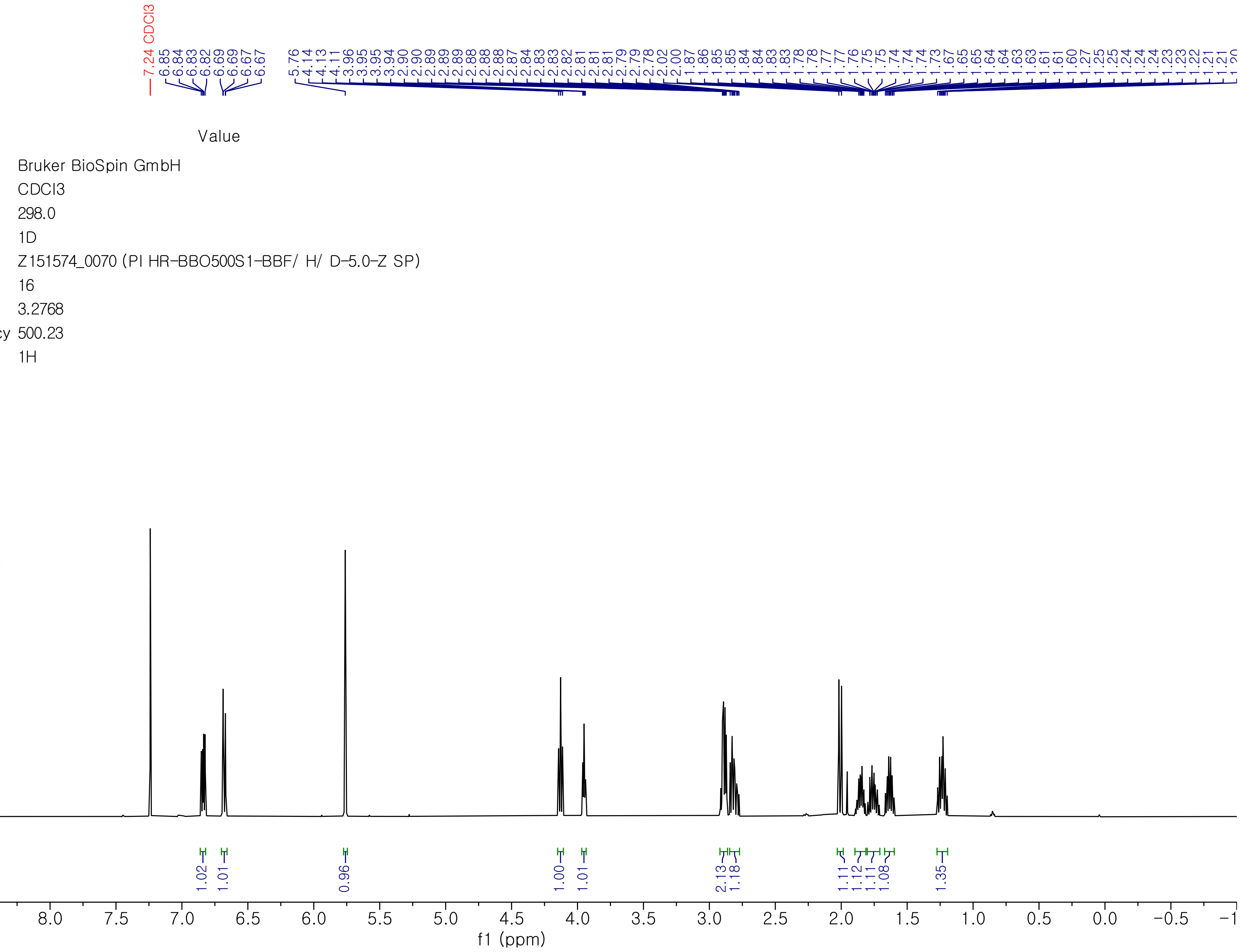

	Parameter	Value
1	Origin	Bruker BioSpin GmbH
2	Solvent	CDCl3
3	Temperature	298.0
4	Experiment	1D
5	Probe	Z151574_0070 (PI HR-BBO500S1-BBF/ H/ D-5.0-Z SP)
6	Number of Scans	16
7	Acquisition Time	3.2768
8	Spectrometer Frequency	500.23
9	Nucleus	1H

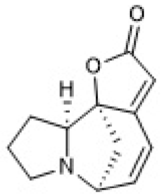

allonorsecurinine (3)

Parameter	Value
1 Origin	Bruker BioSpin GmbH
2 Solvent	CDCl3
3 Temperature	298.0
4 Experiment	1D
5 Probe	Z151574_0070 (PI HR-BBO500S1-BBF/ H/ D-5.0-Z SP)
6 Number of Scans	500
7 Acquisition Time	1.0879
8 Spectrometer Frequency	125.80
9 Nucleus	13C

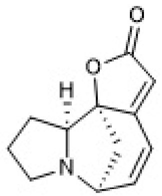

allonorsecurinine (3)

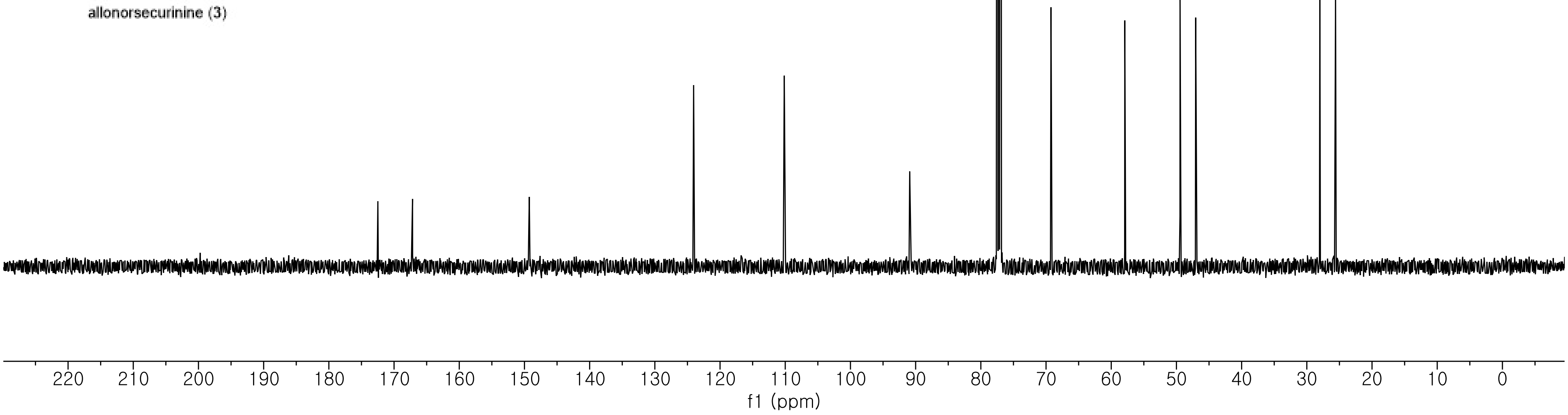

Parameter	Value
1 Origin	Bruker BioSpin GmbH
2 Solvent	CDCl3
3 Temperature	298.0
4 Experiment	1D
5 Probe	Z116098_0402 (PA BBO 400S1 BBF-H-D-05 Z SP)
6 Number of Scans	16
7 Acquisition Time	4.0894
8 Spectrometer Frequency	400.12
9 Nucleus	1H

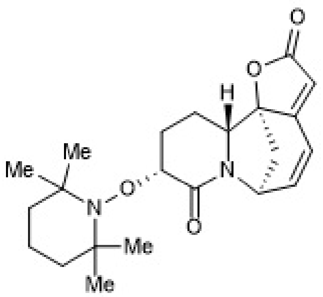

28
(major diastereomer)

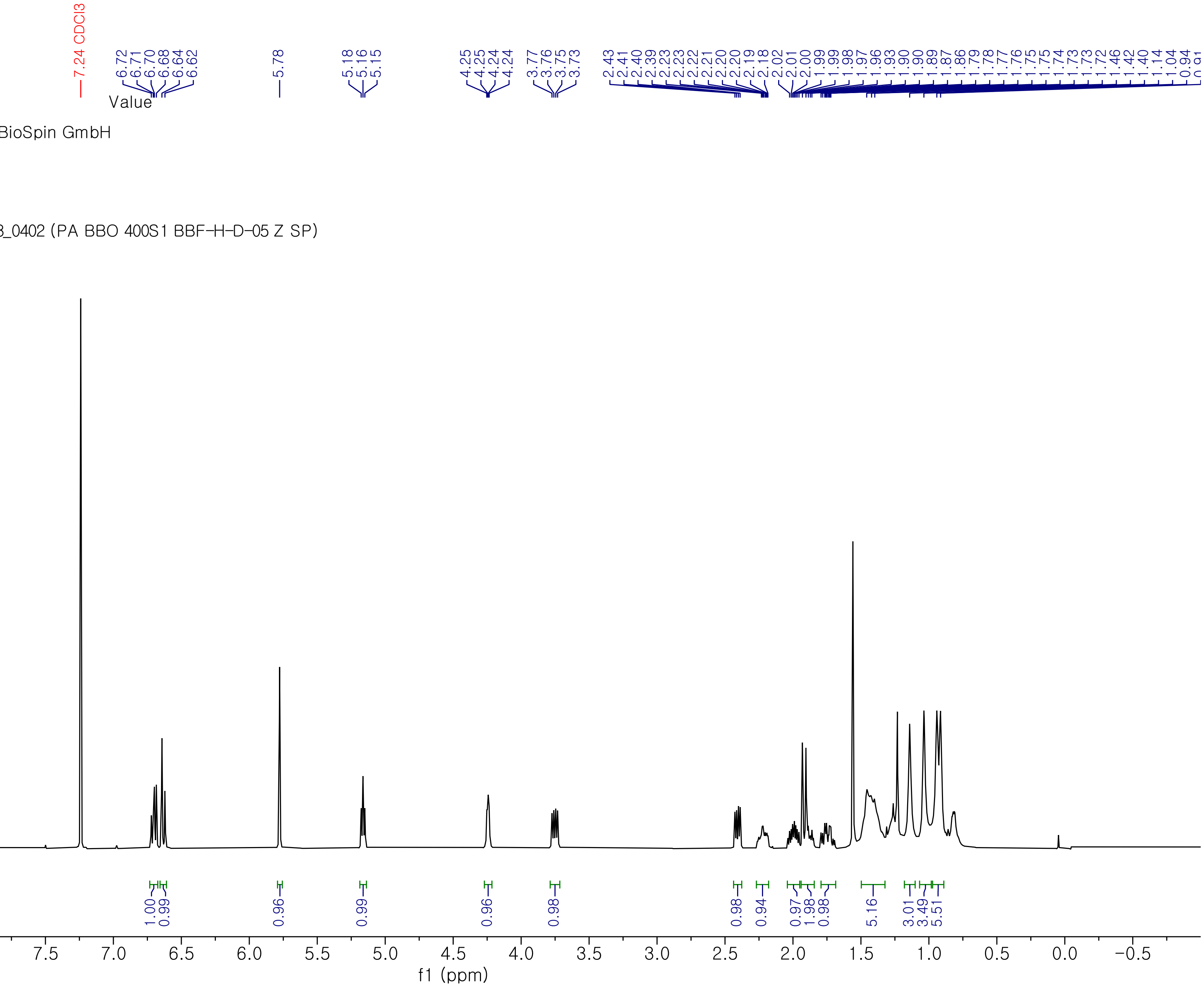

Parameter	Value
1 Origin	Bruker BioSpin GmbH
2 Solvent	CDCl3
3 Temperature	298.0
4 Experiment	1D
5 Probe	Z116098_0402 (PA BBO 400S1 BBF-H-D-05 Z SP)
6 Number of Scans	3072
7 Acquisition Time	1.3631
8 Spectrometer Frequency	100.62
9 Nucleus	13C

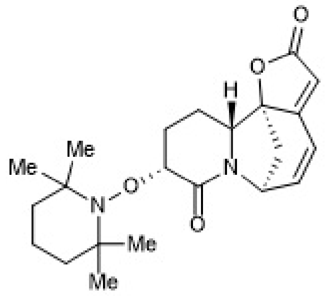

28
(major diastereomer)

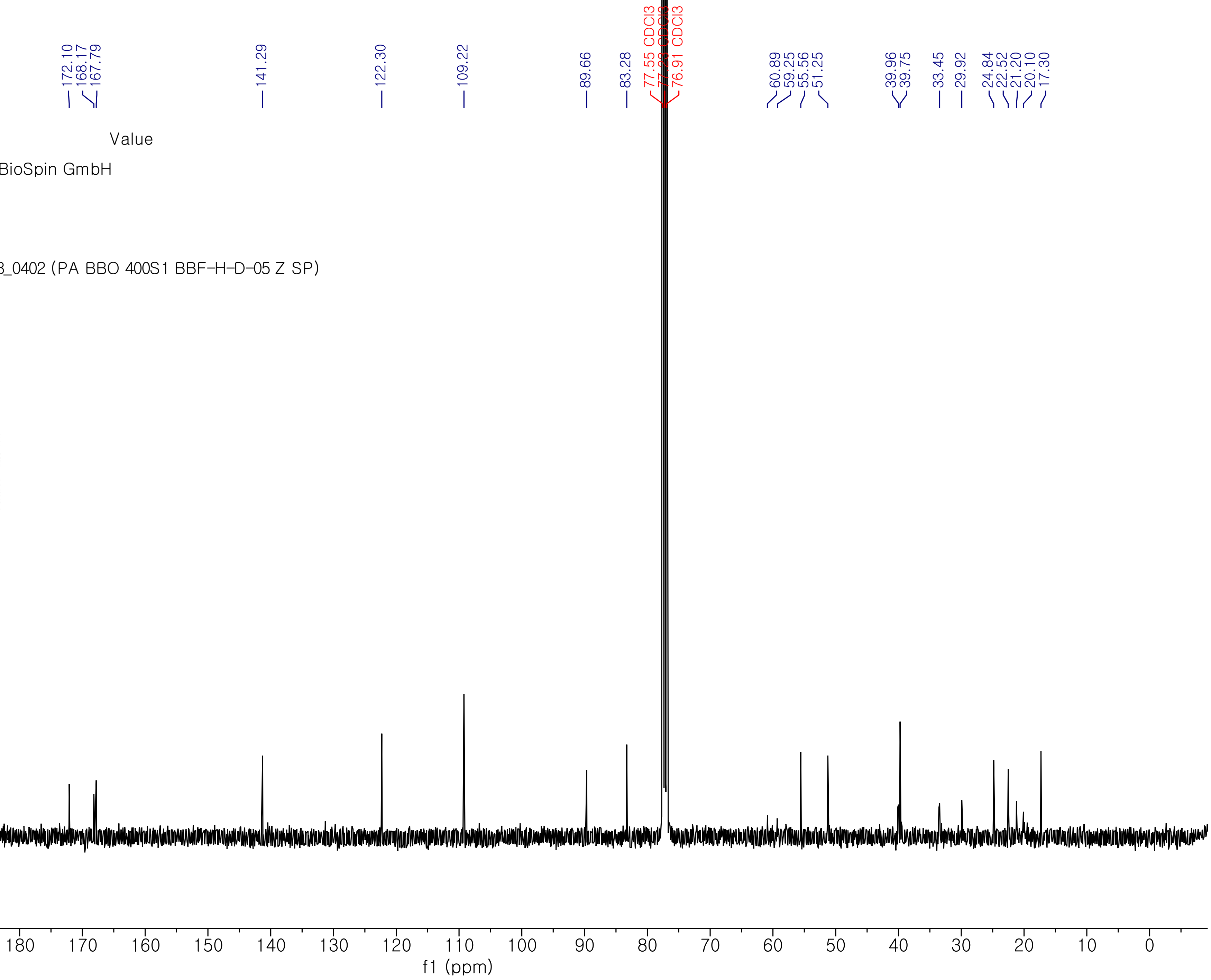

Parameter	Value
1 Origin	Bruker BioSpin GmbH
2 Solvent	CDCl3
3 Temperature	298.0
4 Experiment	1D
5 Probe	Z116098_0402 (PA BBO 400S1 BBF-H-D-05 Z SP)
6 Number of Scans	16
7 Acquisition Time	4.0894
8 Spectrometer Frequency	400.12
9 Nucleus	1H

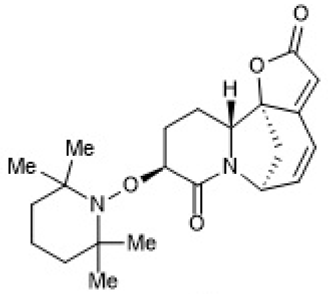

28
(minor diastereomer)

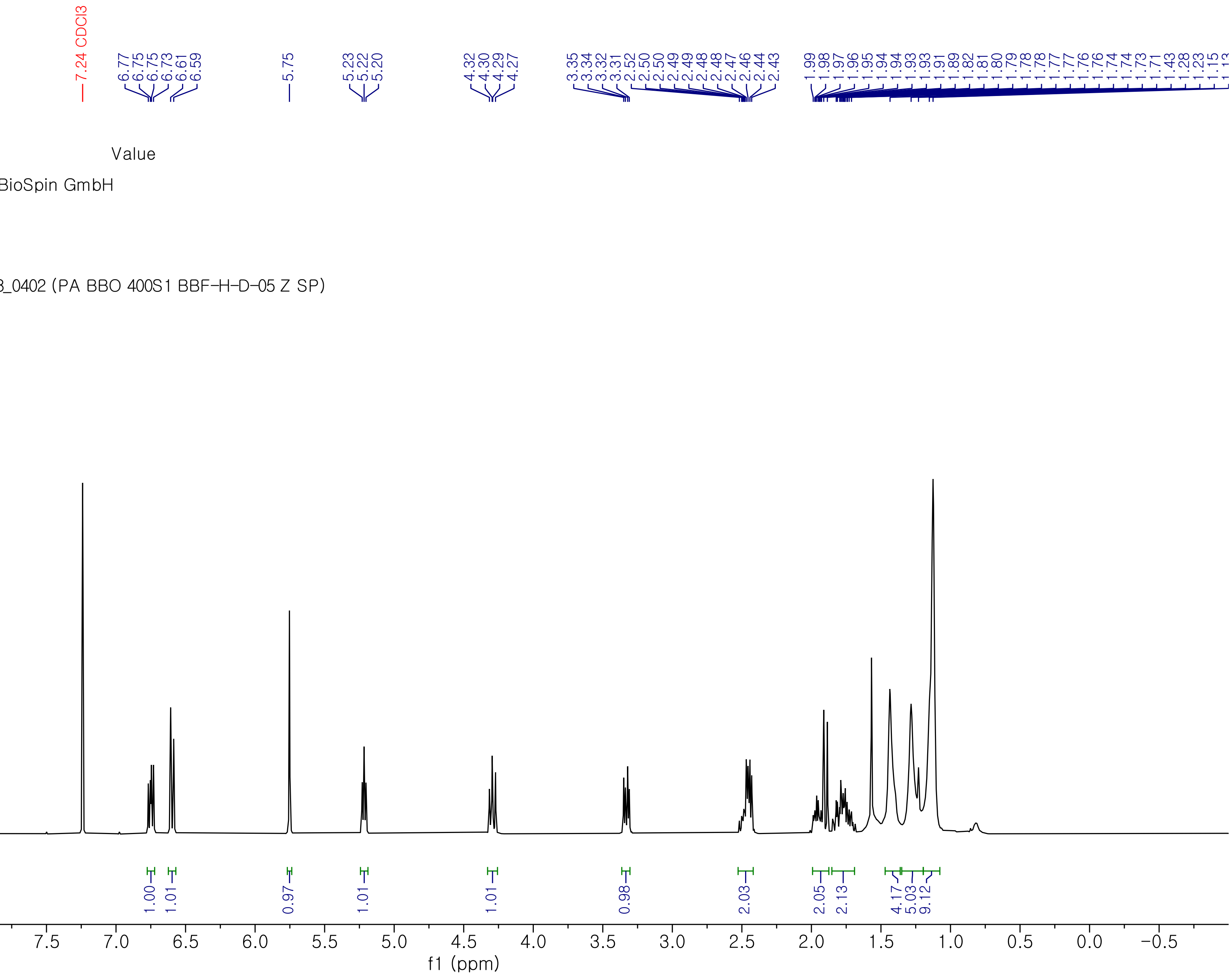

Parameter	Value
1 Origin	Bruker BioSpin GmbH
2 Solvent	CDCl3
3 Temperature	298.0
4 Experiment	1D
5 Probe	Z116098_0402 (PA BBO 400S1 BBF-H-D-05 Z SP)
6 Number of Scans	3072
7 Acquisition Time	1.3631
8 Spectrometer Frequency	100.62
9 Nucleus	13C

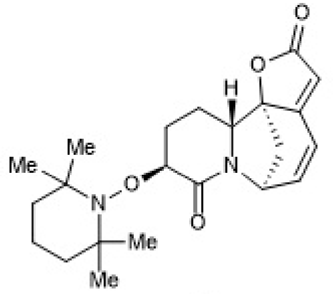

28
(minor diastereomer)

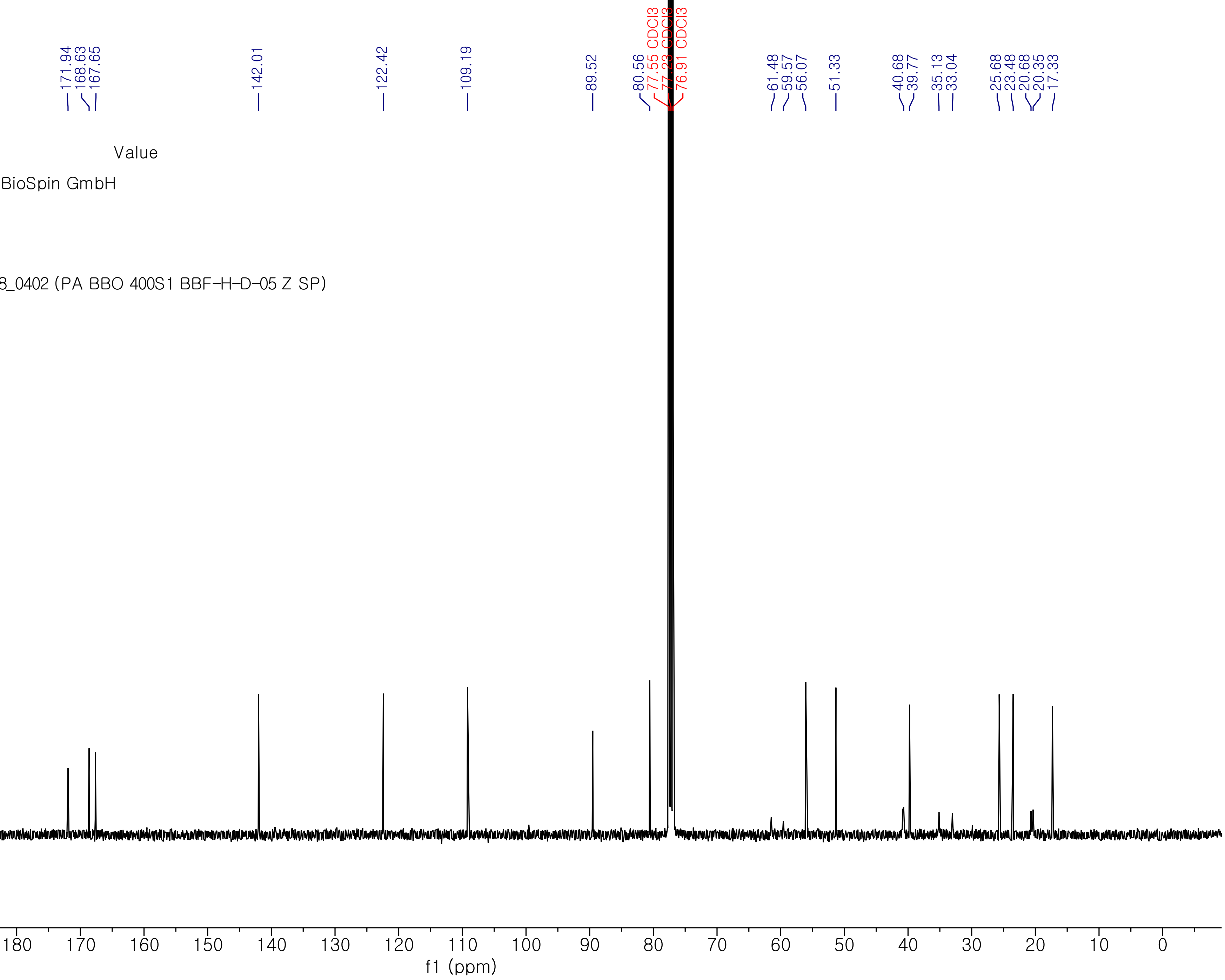

	Parameter	Value
1	Origin	Bruker BioSpin GmbH
2	Solvent	CDCl3
3	Temperature	298.0
4	Experiment	1D
5	Probe	Z116098_0402 (PA BBO 400S1 BBF-H-D-05 Z SP)
6	Number of Scans	16
7	Acquisition Time	4.0894
8	Spectrometer Frequency	400.12
9	Nucleus	1H

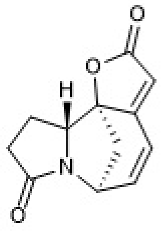

S1

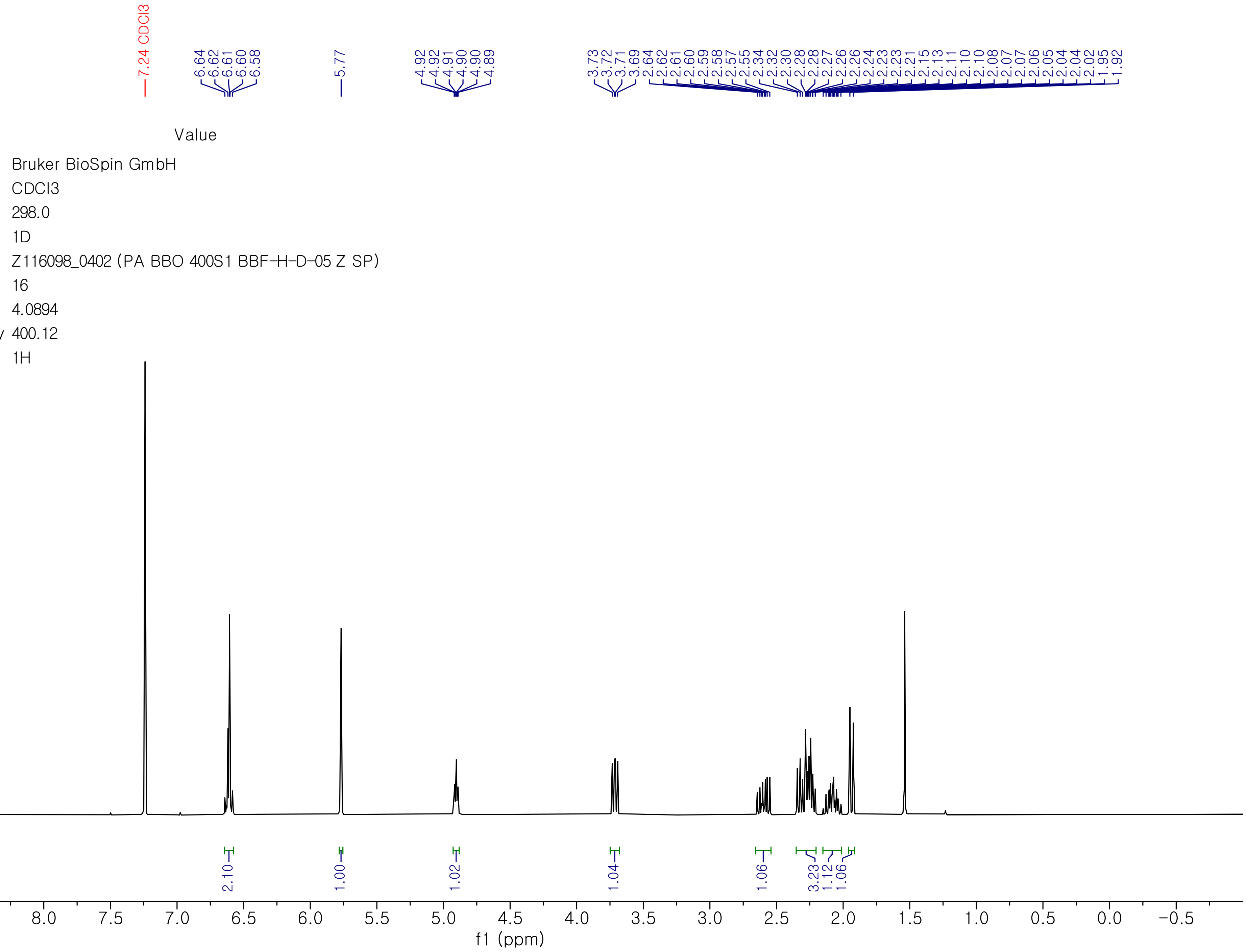

Parameter	Value
1 Origin	Bruker BioSpin GmbH
2 Solvent	CDCl3
3 Temperature	298.0
4 Experiment	1D
5 Probe	Z116098_0402 (PA BBO 400S1 BBF-H-D-05 Z SP)
6 Number of Scans	3072
7 Acquisition Time	1.3631
8 Spectrometer Frequency	100.62
9 Nucleus	13C

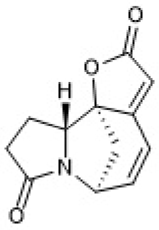

S1

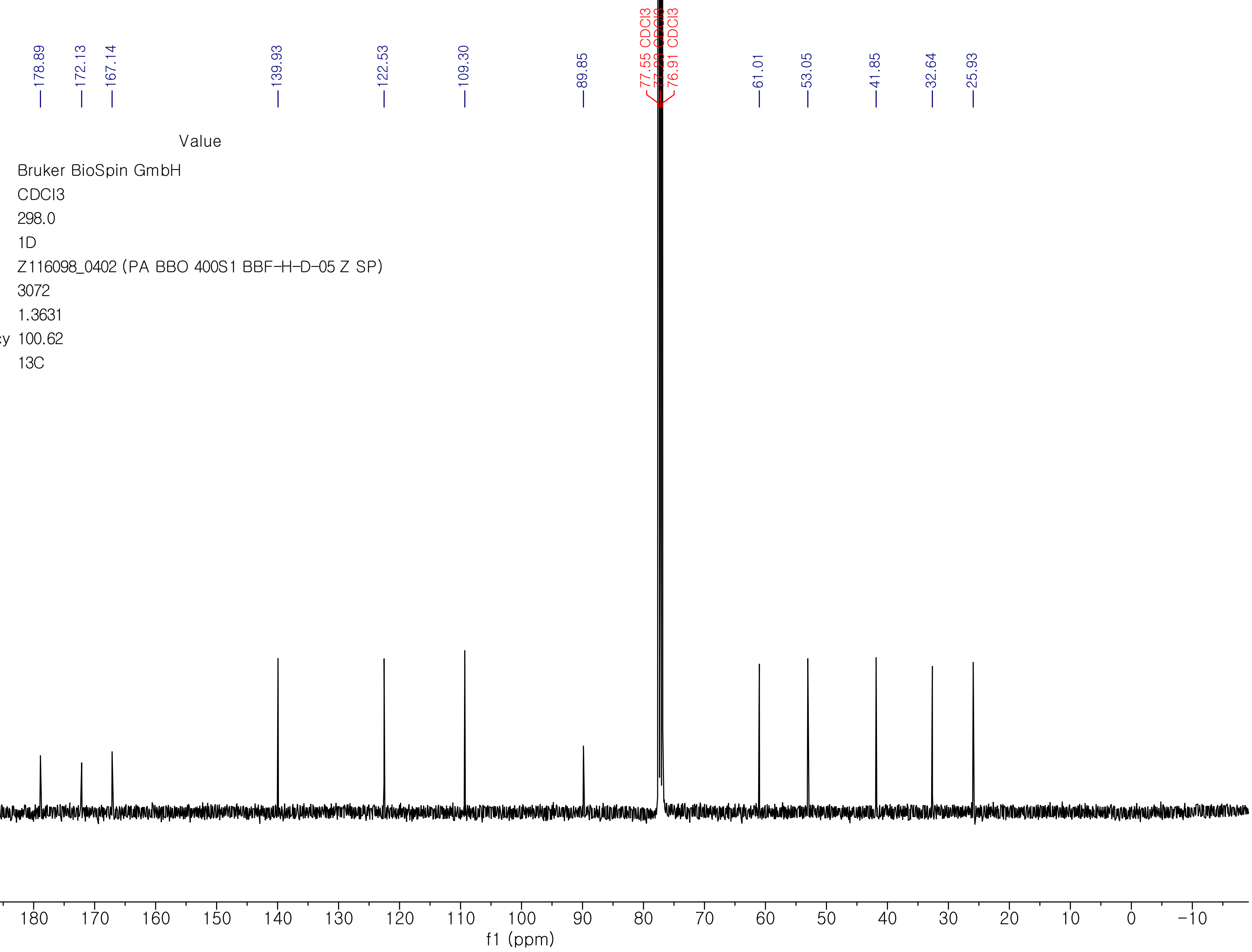

Parameter	Value
1 Origin	Bruker BioSpin GmbH
2 Solvent	CDCl3
3 Temperature	298.0
4 Experiment	1D
5 Probe	Z116098_0402 (PA BBO 400S1 BBF-H-D-05 Z SP)
6 Number of Scans	16
7 Acquisition Time	4.0894
8 Spectrometer Frequency	400.12
9 Nucleus	1H

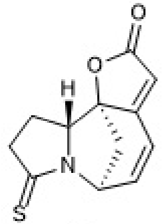

29

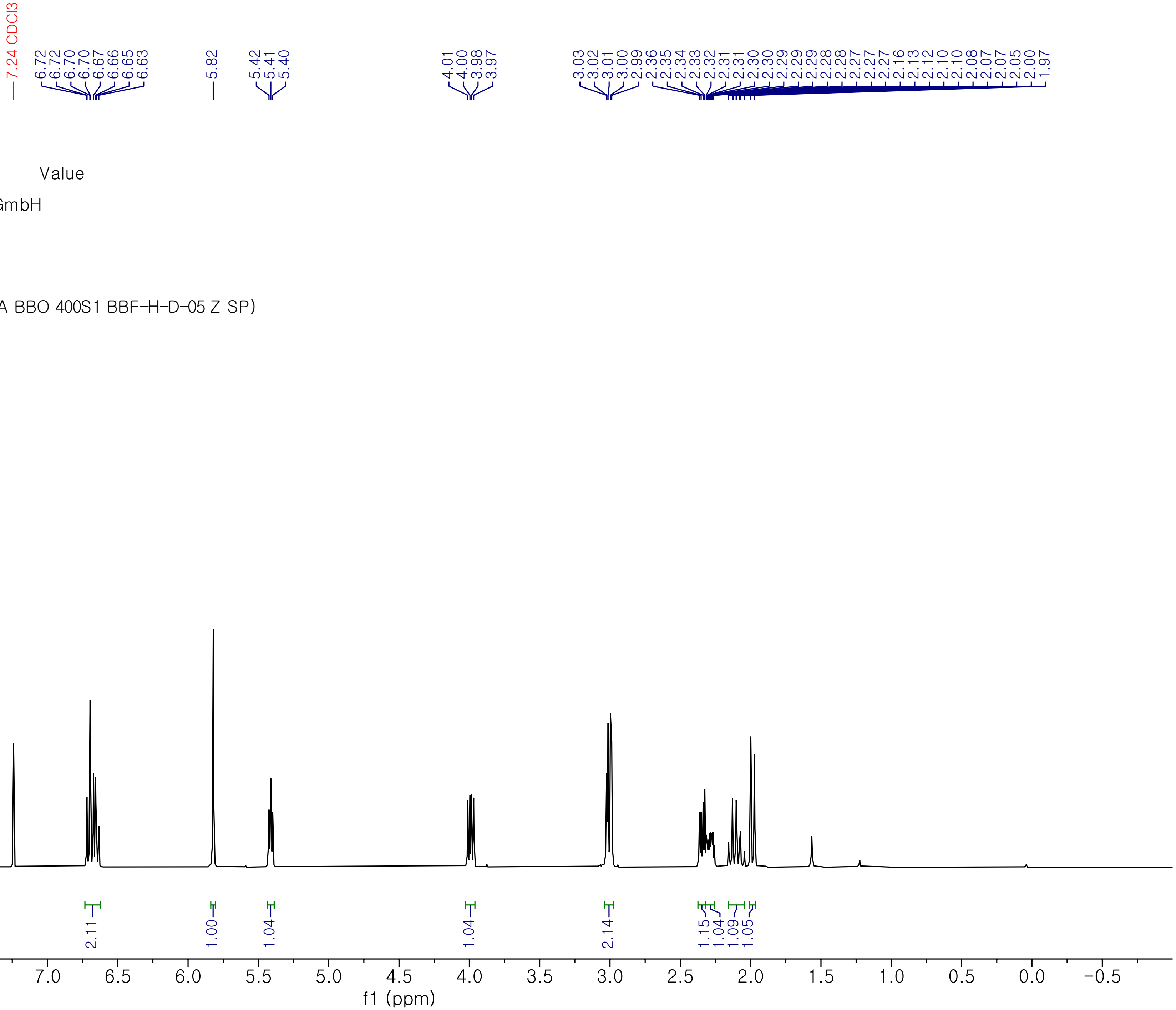

Parameter	Value
1 Origin	Bruker BioSpin GmbH
2 Solvent	CDCl3
3 Temperature	298.0
4 Experiment	1D
5 Probe	Z116098_0402 (PA BBO 400S1 BBF-H-D-05 Z SP)
6 Number of Scans	2048
7 Acquisition Time	1.3631
8 Spectrometer Frequency	100.62
9 Nucleus	13C

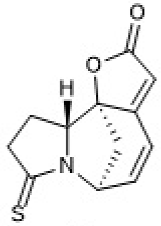

29

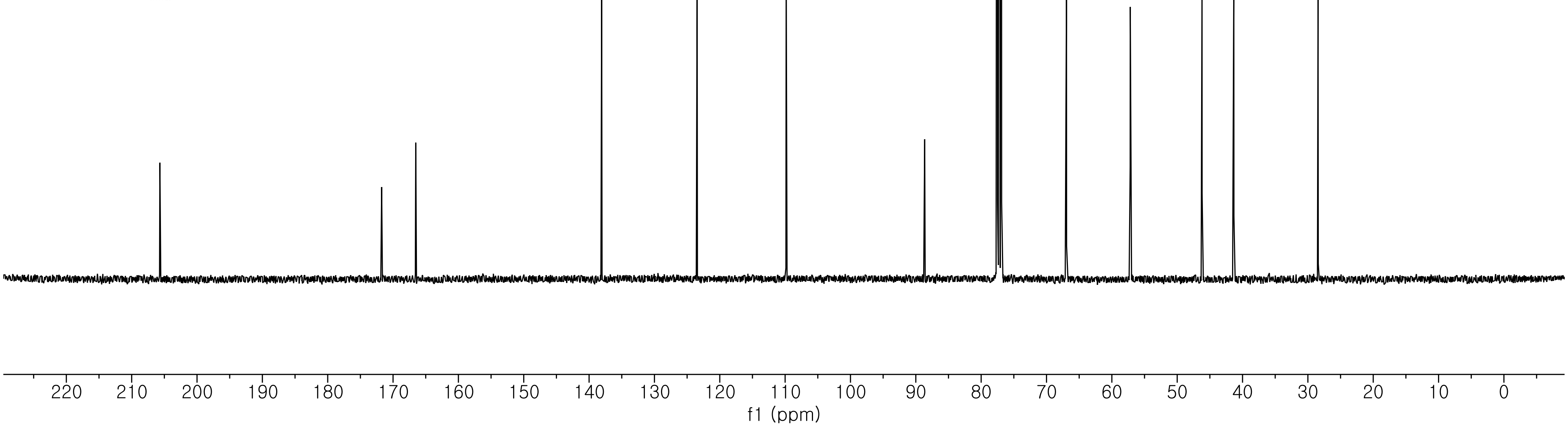

Parameter	Value
1 Origin	Bruker BioSpin GmbH
2 Solvent	CDCl3
3 Temperature	298.0
4 Experiment	1D
5 Probe	Z116098_0402 (PA BBO 400S1 BBF-H-D-05 Z SP)
6 Number of Scans	16
7 Acquisition Time	4.0894
8 Spectrometer Frequency	400.12
9 Nucleus	1H

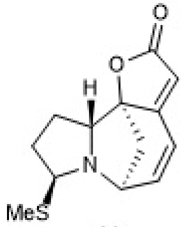

30

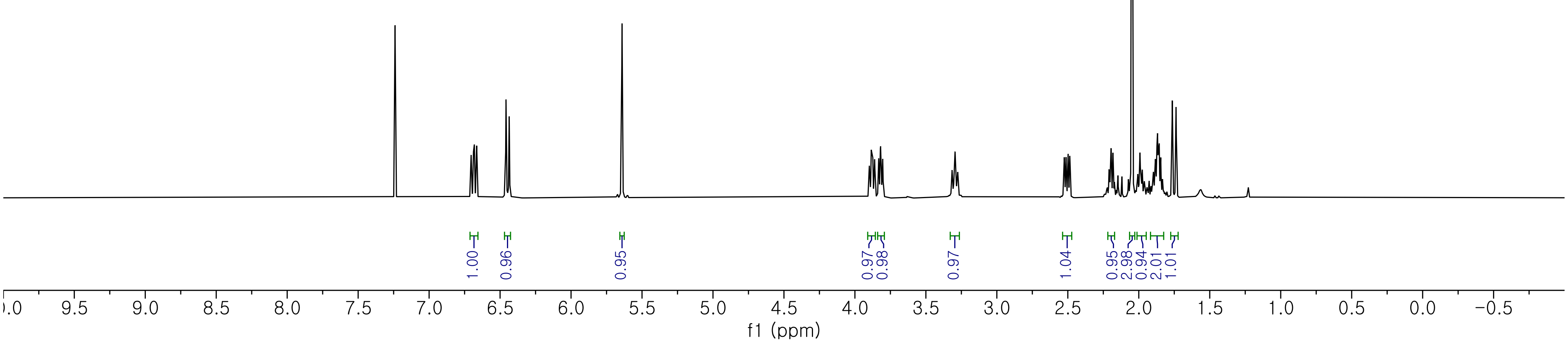

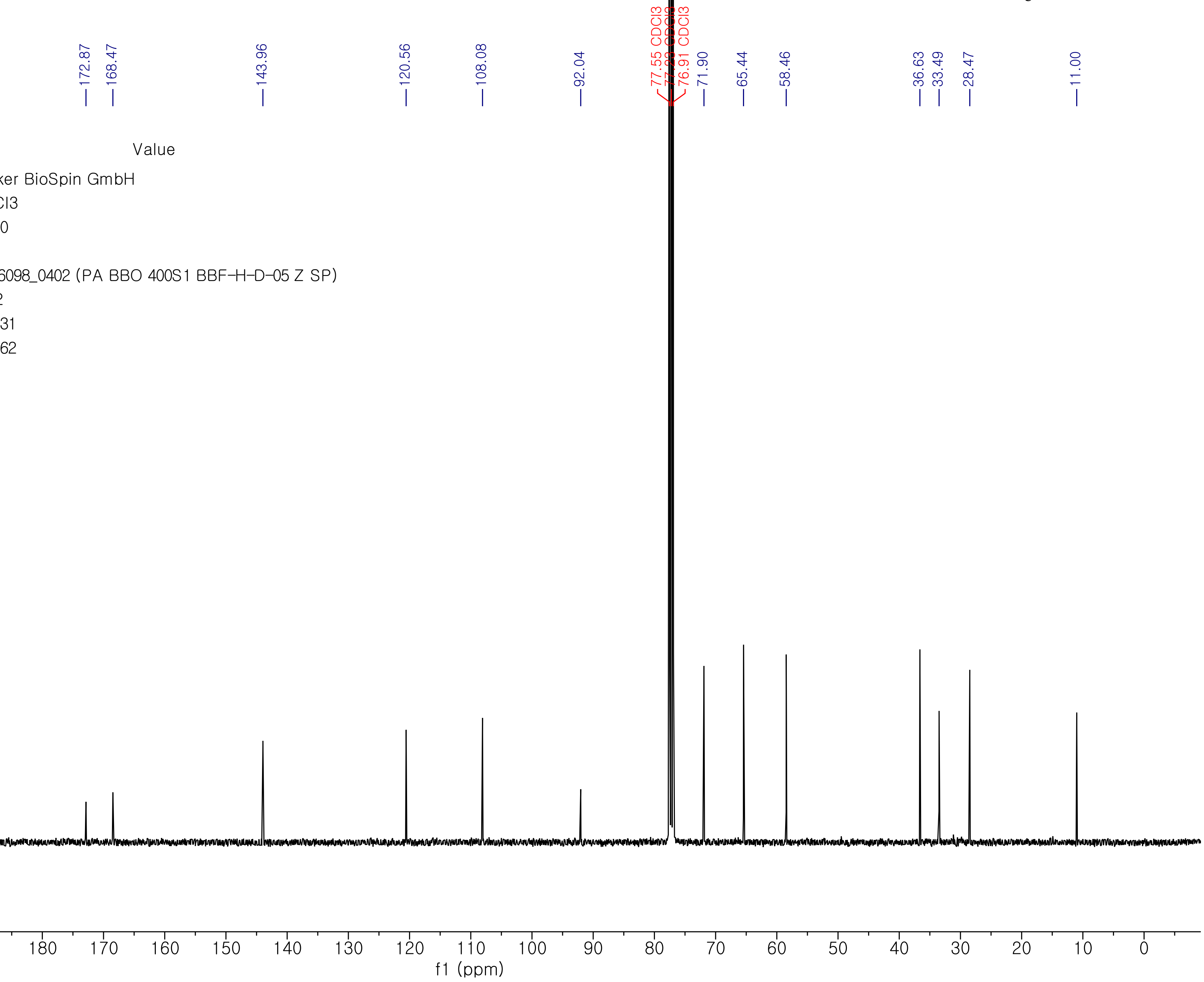

	Parameter	Value
1	Origin	Bruker BioSpin GmbH
2	Solvent	CDCl3
3	Temperature	298.0
4	Experiment	1D
5	Probe	Z116098_0402 (PA BBO 400S1 BBF-H-D-05 Z SP)
6	Number of Scans	3072
7	Acquisition Time	1.3631
8	Spectrometer Frequency	100.62
9	Nucleus	13C

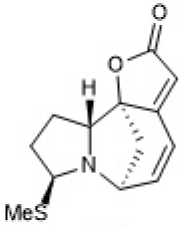

30

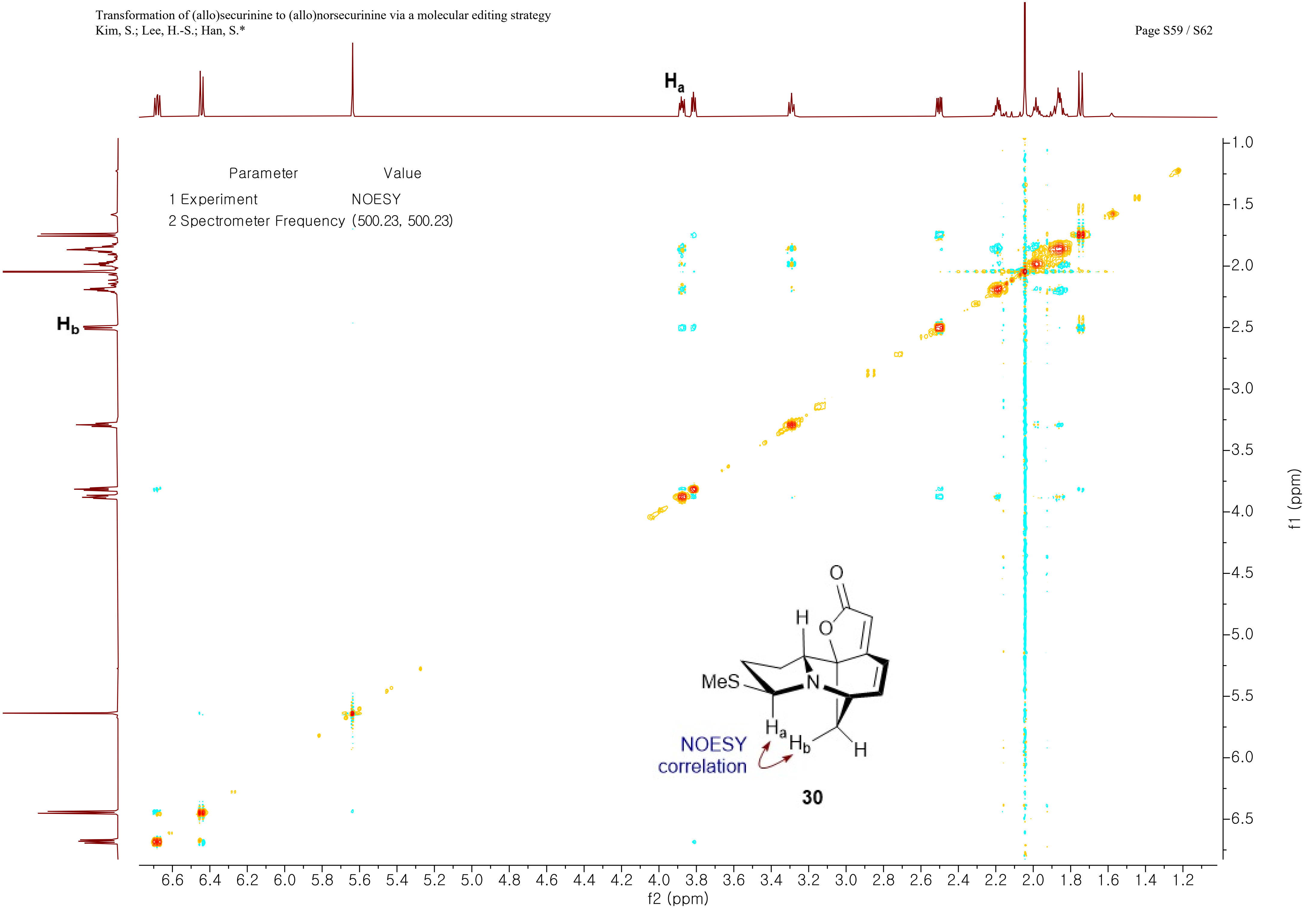
Ha
Hb
Parameter
Value
1 Experiment
NOESY
2 Spectrometer Frequency
(500.23, 500.23)
MeS
N
O
H
Ha
Hb
H
NOESY
correlation
30
f2 (ppm)
f1 (ppm)

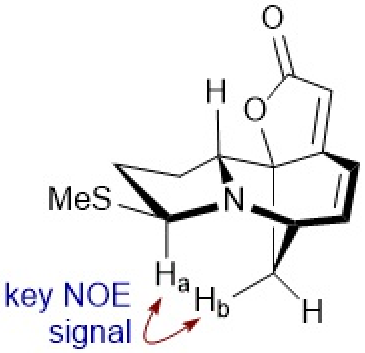
MeS
N
O
O
H
H
Ha
Hb
key NOE signal

30

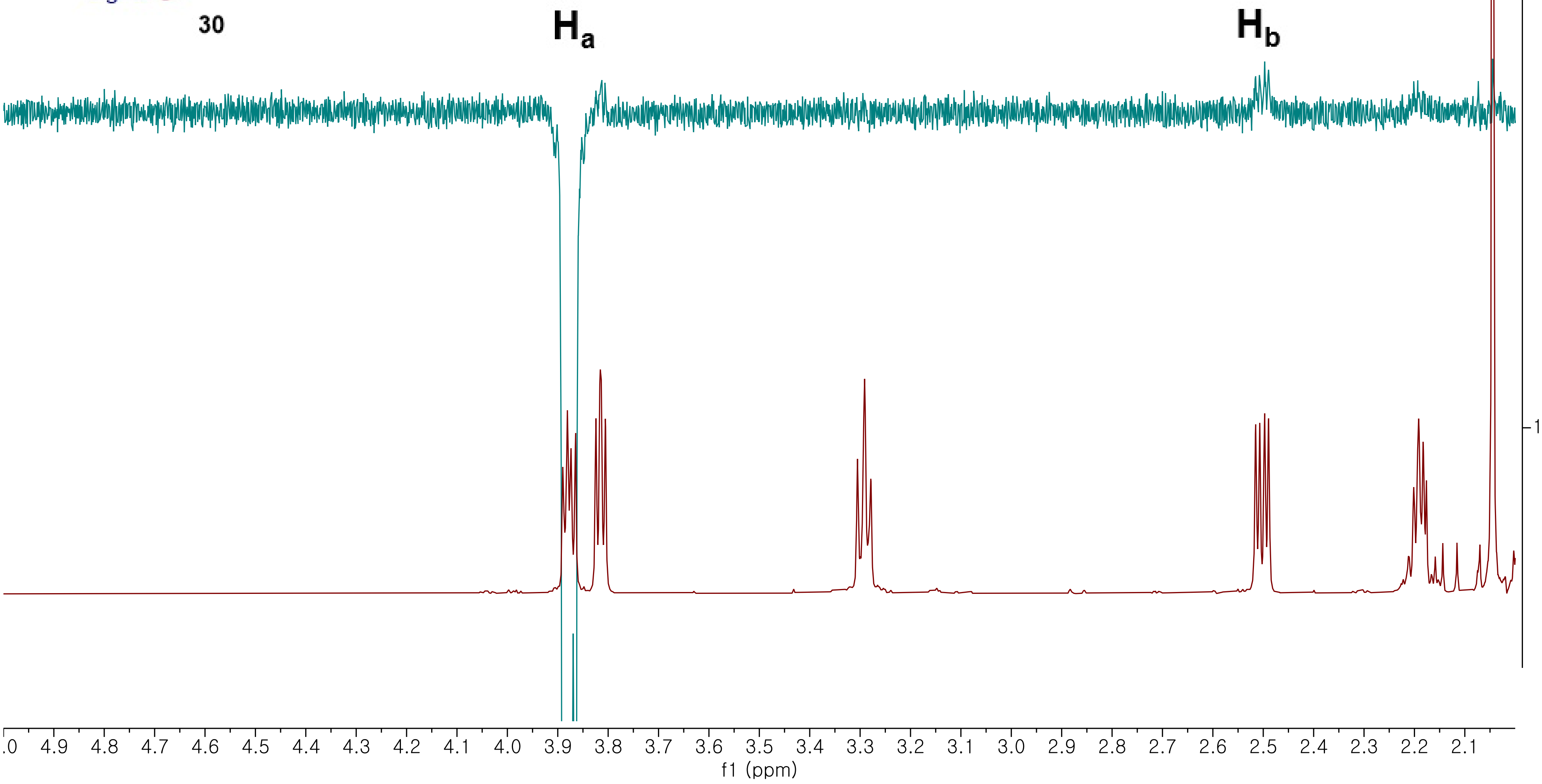
Ha
Hb
5.0
4.9
4.8
4.7
4.6
4.5
4.4
4.3
4.2
4.1
4.0
3.9
3.8
3.7
3.6
3.5
3.4
3.3
3.2
3.1
3.0
2.9
2.8
2.7
2.6
2.5
2.4
2.3
2.2
2.1
f1 (ppm)
2
1

	Parameter	Value
1	Origin	Bruker BioSpin GmbH
2	Solvent	CDCl3
3	Temperature	298.0
4	Experiment	1D
5	Probe	Z116098_0402 (PA BBO 400S1 BBF-H-D-05 Z SP)
6	Number of Scans	16
7	Acquisition Time	4.0894
8	Spectrometer Frequency	400.12
9	Nucleus	1H

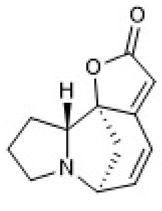

norsecurinine (4)

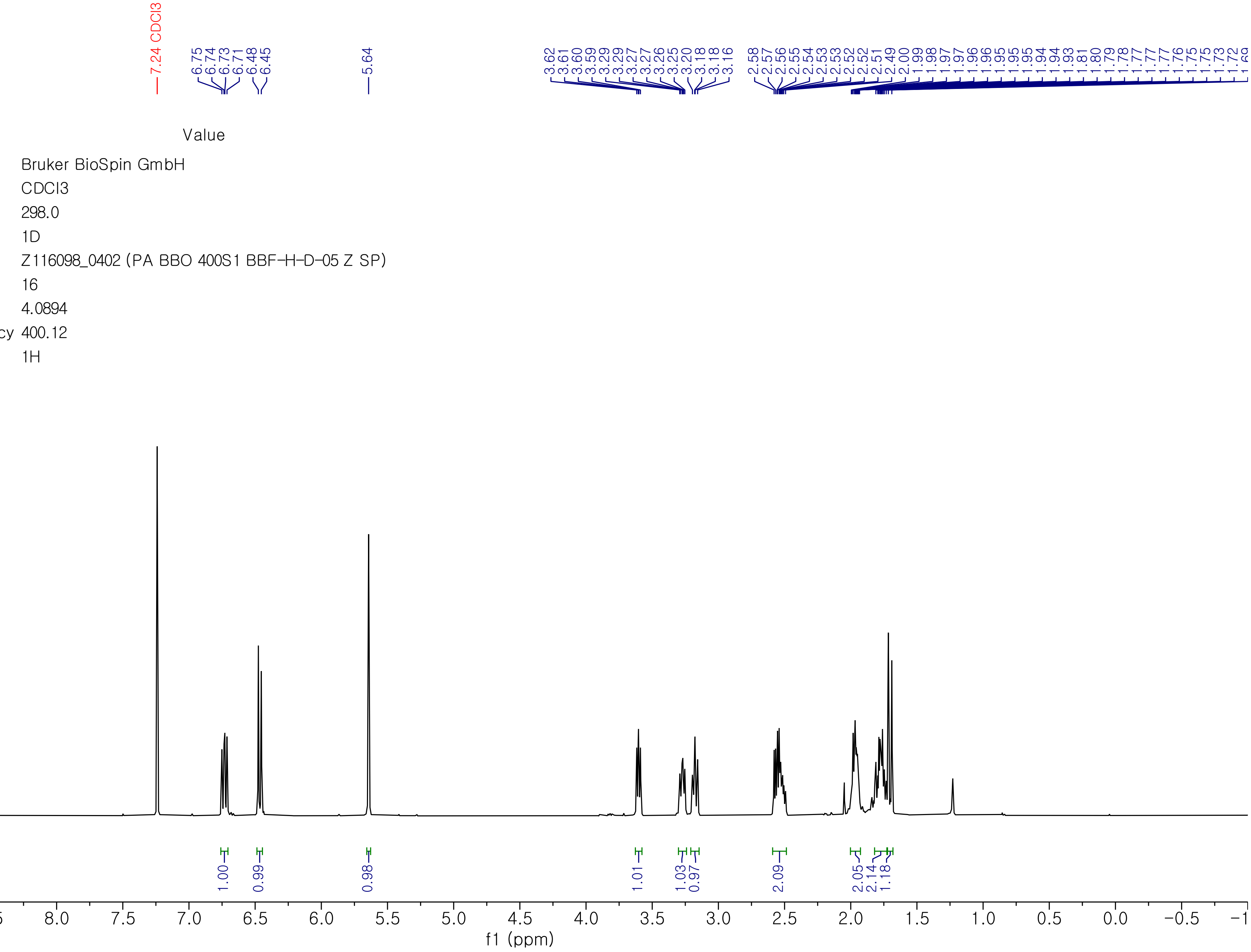

Parameter	Value
1 Origin	Bruker BioSpin GmbH
2 Solvent	CDCl3
3 Temperature	298.0
4 Experiment	1D
5 Probe	Z116098_0402 (PA BBO 400S1 BBF-H-D-05 Z SP)
6 Number of Scans	3072
7 Acquisition Time	1.3631
8 Spectrometer Frequency	100.62
9 Nucleus	13C

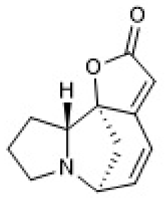

norsecurinine (4)

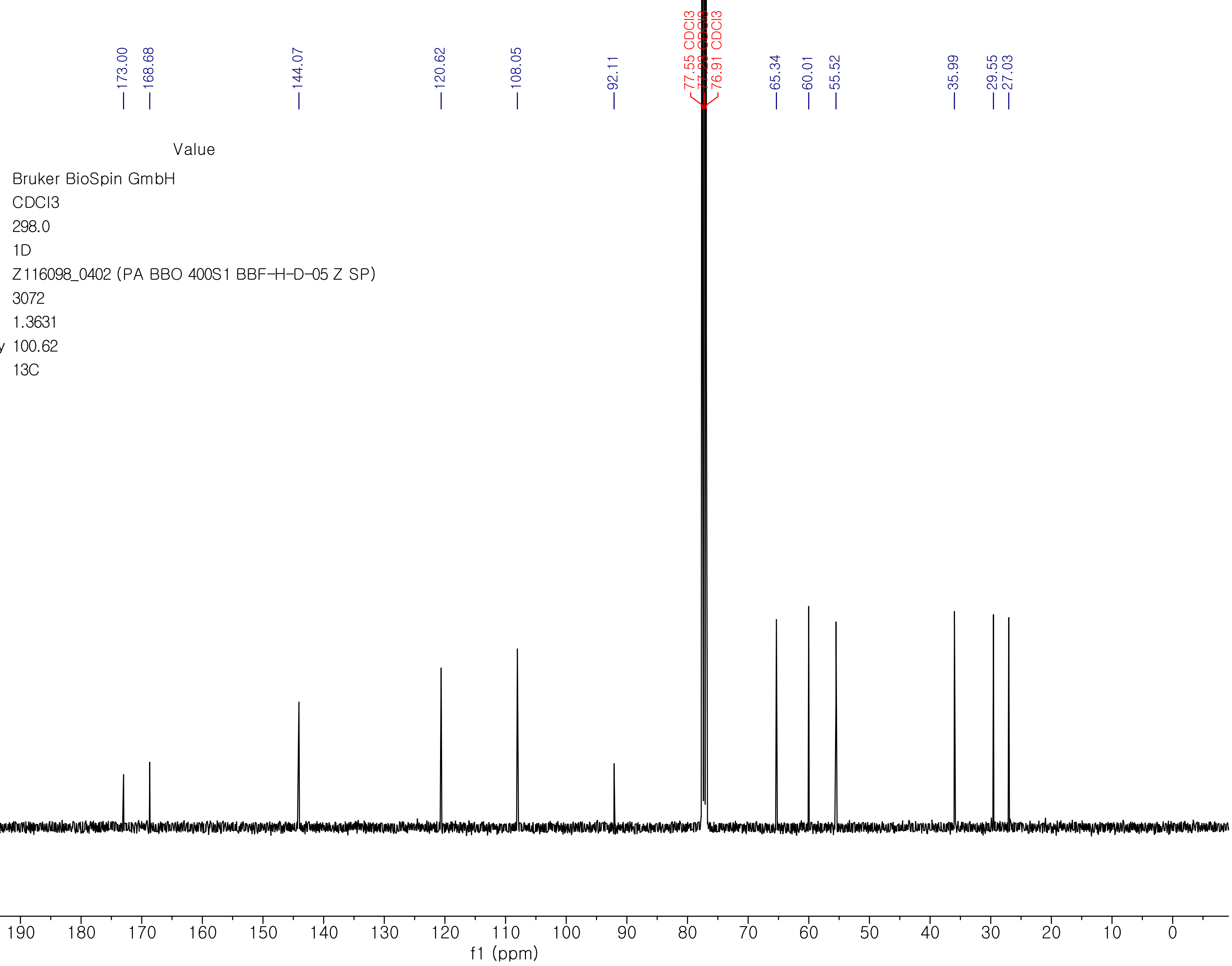

Supplement: Supplementary file 1 [file DataSheet1.pdf]
